# Antitumorigenic potential of *Lactobacillus*-derived extracellular vesicles: p53 succinylation and glycolytic reprogramming in intestinal epithelial cells via SIRT5 modulation

**DOI:** 10.1007/s10565-024-09897-y

**Published:** 2024-08-07

**Authors:** Jingbo Zhang, Xiumei Huang, Tingting Zhang, Chongqi Gu, Wei Zuo, Lijuan Fu, Yiping Dong, Hao Liu

**Affiliations:** 1 Department of Spleen and Stomach Disease, Yubei District Hospital of Traditional Chinese Medicine, Chongqing, 401120 China; 2 Department of Digestion, Rongchang District People’s Hospital of Chongqing, No.3, North Guangchang Road, Changyuan Street, Rongchang District, Chongqing, 402460 China; 3 Department of Pediatrics, Rongchang District People’s Hospital, Chongqing, 402460 China; 4 Department of Herbal Medicine, School of Traditional Chinese Medicine, Chongqing Medical University, Chongqing, 400016 China; 5 Department of Pharmacology, Academician Workstation, Changsha Medical University, Changsha, 410219 China; 6 Department of Digital Medicine, Department of Bioengineering and Imaging, Army Medical University, Chongqing, 400038 China

**Keywords:** Colorectal cancer, *Lactobacillus*, Extracellular vesicles, SIRT5, p53, Palmitoylation modification

## Abstract

**Objective:**

Colorectal cancer progression involves complex cellular mechanisms. This study examines the effects of *Lactobacillus plantarum*-derived extracellular vesicles (LEVs) on the SIRT5/p53 axis, focusing on glycolytic metabolic reprogramming and abnormal proliferation in intestinal epithelial cells.

**Methods:**

LEVs were isolated from *Lactobacillus plantarum* and incubated with Caco-2 cells. Differential gene expression was analyzed through RNA sequencing and compared with TCGA-COAD data. Key target genes and pathways were identified using PPI network and pathway enrichment analysis. Various assays, including RT-qPCR, EdU staining, colony formation, flow cytometry, and Western blotting, were used to assess gene expression, cell proliferation, and metabolic changes. Co-immunoprecipitation confirmed the interaction between SIRT5 and p53, and animal models were employed to validate *in vivo* effects.

**Results:**

Bioinformatics analysis indicated the SIRT5/p53 axis as a critical pathway in LEVs' modulation of colorectal cancer. LEVs were found to inhibit colorectal cancer cell proliferation and glycolytic metabolism by downregulating SIRT5, influencing p53 desuccinylation. In vivo, LEVs regulated this axis, reducing tumor formation in mice. Clinical sample analysis showed that SIRT5 and p53 succinylation levels correlated with patient prognosis.

**Conclusion:**

*Lactobacillus*-derived extracellular vesicles play a pivotal role in suppressing colonic tumor formation by modulating the SIRT5/p53 axis. This results in decreased glycolytic metabolic reprogramming and reduced proliferation in intestinal epithelial cells.

**Supplementary Information:**

The online version contains supplementary material available at 10.1007/s10565-024-09897-y.

## Introduction

Colorectal cancer is a malignancy with relatively high incidence and mortality rates globally (Lu et al. 2021; Roslan et al. 2019; Otani et al. 2019; Islam et al. 2022). The difficulty in treating this condition arises from its early symptoms being not readily apparent, causing many patients to already reach the middle and late stages when diagnosed (Lahmidani et al. 2019; Fabregas et al. 2022; Aiello et al. 2019). In recent years, extensive research has revealed an association between the gut microbiota and the occurrence and progression of tumors (Qin et al. 2024; Jackson and Theiss 2020; Song et al. 2020). *Lactobacillus*, as beneficial bacteria in the intestines, have been increasingly studied for their potential role in preventing and treating colon cancer (Ma et al. 2022; Lightfoot et al. 2013).

Probiotics aid in maintaining the balance of the intestinal tract and preventing excessive growth of harmful bacteria. Additionally, they produce various bioactive substances like lactic acid and casein, which possess multiple functions, including antioxidant, anti-inflammatory, and anti-tumor properties (Wieërs et al. 2020; Chee et al. 2020; Naureen et al. 2022). In recent times, extracellular vesicles (EVs) derived from *Lactobacillus* have garnered the interest of researchers as a novel means of intercellular communication (Briaud and Carroll 2020). Research has discovered that LEVs are abundant in diverse bioactive compounds, potentially contributing to human health and disease development (Kim et al. 2022).

Metabolic reprogramming refers to the process through which tumor cells adapt their energy metabolism to meet the demands of their rapid proliferation and growth (Dai et al. 2020). Glycolytic metabolic reprogramming is the predominant mode of metabolic reprogramming in tumor cells, resulting in elevated glucose uptake and the generation of a quantity of lactate (Liu et al. 2023; Chen et al. 2022). Furthermore, the ability of colon cancer cells to proliferate is closely linked to their metabolic status (Chen and Shen 2020). Numerous studies have shown that metabolic reprogramming plays a crucial role in the initiation and progression of tumors, potentially serving as a novel predictive biomarker for tumor immunotherapy response (Sung and Cheong 2021, 2022a, b). Consequently, the reprogramming and increased proliferation of glycolytic metabolism in intestinal epithelial cells has emerged as a crucial area of focus for treating colon cancer.

Both SIRT5 and p53 play essential roles as regulatory factors in cell growth, metabolism, and survival (Das 2021). Recent studies have demonstrated that SIRT5 controls cellular metabolism and viability through desuccinylation modifications on diverse substrates (Kwon et al. 2023). p53, a tumor suppressor protein, has its activity and stability regulated through diverse post-translational modifications, including acetylation (Antropova et al. 2022). Research on both SIRT5 and p53 has separately indicated associations with the initiation and progression of colon cancer (Miura et al. 2021). However, the relationship between SIRT5 and p53, along with their roles in colon cancer, remains unclear, necessitating further investigation for elucidation. Nonetheless, the association between SIRT5 and p53 and their involvement in colorectal cancer remains uncertain, necessitating additional research for clarification.

In conclusion, thoroughly investigating the role of *Lactobacillus*-derived outer membrane vesicles in regulating glycolytic metabolism reprogramming and abnormal proliferation in intestinal epithelial cells through SIRT5-mediated p53 succinylation is of great significance. This research will offer novel strategies for unveiling colon cancer's pathogenesis, prevention, and treatment.

## Materials and methods

### Ethics statement

This study was carried out with the approval and oversight of the Institutional Review Board of Rongchang District People’s Hospital. The animal experimental procedures were approved by the Animal Ethics Committee. The clinical aspects of this research involving human participants were reviewed and approved by the Clinical Ethics Committee with the ethical. Informed consent was obtained from all subjects.

### *Lactobacillus Plantarum* was cultivated, and its outer membrane vesicles were extracted, isolated, and identified

The strain of *L. plantarum* (Bio-67374) we obtained was derived from plants and sourced from the Chinese Microbial Strain Resource Database. A 1% inoculum was added to 10 mL of sterilized MRS broth (CM0359B, Thermo Fisher Scientific, Waltham, MA, USA), followed by incubation at 37℃ for 24 h. Subsequently, the bacterial suspension was mixed with 50% glycerol at a 1:1 ratio and stored frozen at -80 °C. To assess growth and pH variations, 5% *Lactobacillus plantarum* was inoculated into MRS broth and cultured at 37 °C for 48 h, with bacterial counts measured at 0, 8, 24, and 48 h. Bacterial counts were measured at 0, 8, 24, and 48 h. The formula for calculating the bacterial colony count is obtained by dividing the colony count by the dilution volume and then multiplying the result by the dilution factor. The samples are collected at 0, 2, 8, 16, 24, and 48 h. Subsequently, the pH of the culture medium is measured using a pH meter.

Extraction and separation of LEVs: *L. plantarum* was cultured until it reached the stationary phase with colony forming units (CFU) of ≥ 2.7 × 10^9^ CFU/mL *L. plantarum*. The culture was then subjected to consecutive centrifugation at 1800 × g at 4 °C for 20 min and 10,000 × g at 4 °C for 20 min to collect the bacterial pellet and supernatant, respectively. Filtering was conducted using a cellulose acetate membrane (HAWG0470, Millipore, Merck, UK) with a pore size of 0.45 μm. Concentration was accomplished using a Centricon Plus-70 membrane Ultracel-PL (UFC710008, Millipore, Merck, UK) with a molecular weight cut-off of 100 kDa. The concentrated supernatant should be centrifuged at 4 °C and 260,000 × g for one hour. After that, the pellet should be washed twice with phosphate-buffered saline (PBS) or 0.1 M salt-free phosphate buffer (PB). The EV-containing pellet should be resuspended in PBS and stored at -20 °C for further experiments.

To identify LEVs, they are fixed with 1% glutaraldehyde, placed on grids coated with formic acid ester, and restained with 30 μL of 1% phosphotungstic acid solution (pH 6.8) for 5 min at room temperature. Dry the sample using an incandescent lamp and observe it under a JEM-2000EX transmission electron microscope (JEOL, Japan), capturing images at the appropriate magnification. The Zetasizer Nano ZS90 (Malvern, UK) was utilized for quantifying and analyzing the size distribution of extracellular vesicles (EVs) (Kim et al. 2020; Bajic et al. 2020; Lee et al. 2021; Li et al. 2017; Yang et al. 2022).

### Cell culture

We utilized the following human colon cancer cell lines: CaCo-2 (CC-Y1089), SW480 (CC-Y1500), and HCT-116 (CC-Y1194). Additionally, we employed the human normal colon epithelial cell line NCM460 (CC-Y1550) (1) and the human embryonic kidney cell line 293 T (CC-Y1010) (1). All cell lines were acquired from Enzyme Research Biotechnology in Shanghai, China. The CaCo-2 and 293 T cells were cultured in high-glucose DMEM medium (11,965,084, Thermo Fisher Scientific, USA) supplemented with 20% or 10% FBS (26,140,079, Thermo Fisher Scientific, USA) and 1% antibiotic–antimycotic solution (100 U/mL penicillin and 100 μg/mL streptomycin) respectively. The SW480, HCT-116, and NCM460 cells were cultured in Leibovitz's L-15 Medium (11,415,064, Thermo Fisher Scientific, USA), McCoy's 5A Medium (16,600,082, Thermo Fisher Scientific, USA), and RPMI-1640 (11,875,101, Thermo Fisher Scientific, USA) respectively, supplemented with 10% FBS and 1% antibiotic–antimycotic solution. All cell cultures were conducted in a temperature-controlled incubator (model BB15, Thermo Fisher Scientific, USA) at 37 ℃ with 5% CO_2_ (Li et al. 2017).

### Cellular uptake of *Lactobacillus plantarum*-derived extracellular vesicles

The LEVs were labeled with PKH26 green fluorescent dye (MINI26, Sigma-Aldrich, USA) and then incubated at room temperature for 5 min with a concentration of 10 μg/ml of the labeled LEVs (Xian et al. 2019). The cells were suspended in a standard culture medium and incubated with Caco-2 cells at 37 °C for 24 h. Then, wash the cells twice with PBS. DAPI is used to label cell nuclei. Stained cells were observed using the IX53 fluorescence microscope (CLS; LSM 510 META, Carl Zeiss AG) (Cheng et al. 2018; Wei et al. 2020).

### RNA extraction and sequencing

*Lactobacillus plantarum* cells were collected by centrifugation at 12,000 g for 5 min. Then, the cells were suspended in a DMEM high glucose medium supplemented with 1% non-essential amino acids until an optical density of 0.9 at 600 nm was reached. Caco-2 cells were treated with a cell suspension containing *Lactobacillus plantarum* cells or a control medium for 10 h. Subsequently, two groups of cell samples were collected, each containing four samples, and total RNA was isolated using Trizol Reagent (15,596,026, Invitrogen, Thermo Fisher Scientific, Waltham, MA, USA).

The concentration and purity of the RNA samples were measured using a Nanodrop 2000 spectrophotometer (1011U, Nanodrop, Thermo Fisher Scientific, Waltham, MA, USA). Subsequent experiments utilize total RNA samples that meet the following criteria: RNA Integrity Number (RIN) ≥ 7.0 and a 28S:18S ratio ≥ 1.5, determined through denatured agarose gel electrophoresis and Bioanalyzer 2100 analysis for assessing RNA integrity.

CapitalBio Technology, located in Beijing, China, prepared and sequenced the sequencing library. Each sample requires a total of 5 μg of RNA. To eliminate ribosomal RNA (rRNA) from total RNA, the Ribo-Zero™ Magnetic Kit (MRZE706, Epicentre Technologies, Madison, Wisconsin, USA) is employed. The NEB Next Ultra RNA Library Prep Kit (#E7775, NEB, USA) is utilized for Illumina sequencing and library construction. Subsequently, the RNA fragment is fragmented into approximately 300 base pair (bp) fragments using NEB Next First Strand Synthesis Reaction Buffer (5 ×). The first strand of cDNA is synthesized using reverse transcriptase primers and random primers, while the synthesis of the second strand of cDNA takes place in the reaction buffer of dUTP Mix (10 ×) for the second strand synthesis. The repair of cDNA fragment ends involves the addition of a polyA tail and the ligation of sequencing adaptors.

Following the ligation of Illumina sequencing adapters, the second strand of cDNA was digested using USER Enzyme (#M5508, NEB, USA) to construct a strand-specific library. The library DNA should be amplified, followed by purification and enrichment through PCR. Subsequently, the library was analyzed using an Agilent 2100 instrument and quantified utilizing the KAPA Library Quantification Kit (KK4844, KAPA Biosystems). Finally, we conducted paired-end sequencing using the NextSeq CN500 (Illumina) sequencer (Regan et al. 2021; Bao et al. 2023).

### Quality control of the sequencing data and alignment to the reference genome should be performed

FastQC software version 0.11.8 assessed the quality of paired-end reads in the raw sequencing data. The raw data was preprocessed using version 1.18 of the Cutadapt software to eliminate Illumina sequencing adapters and poly(A) tail sequences. We used a Perl script to discard reads with an N content of over 5%. Reads with a base quality of over 20 were extracted, amounting to 70% of the total, using the FASTX Toolkit version 0.0.13 software. Repair the paired-end sequences using BBMap software. The filtered high-quality read fragments were finally aligned to the human reference genome using hisat2 software (version 0.7.12) (Bao et al. 2023).

### Bioinformatics analysis

Transcriptome sequencing data for colon cancer (TCGA-COAD) were obtained from The Cancer Genome Atlas (TCGA) database (https://portal.gdc.cancer.gov/), comprising 483 colon cancer tissue samples and 41 adjacent normal tissue samples. The mRNA expression levels were analyzed using the "edgeR" package in R language (version 4.2.1) based on read counts. Differential expression analysis was performed between Caco-2 cell samples and TCGA-COAD dataset samples, applying the criteria |log2FC|> 0 and P.value < 0.05 for selecting differentially expressed genes.

Colon cancer-related genes were retrieved from disease-related search databases, phenolyzer (https://phenolyzer.wglab.org/) and CTD (https://ctdbase.org/), with filtering criteria set at Score > 0.0015 and Inference Score > 25, respectively. The R packages ggplot2 (version 3.3.6) and VennDiagram (version 1.7.3) were employed to generate and visualize a Venn diagram to obtain the intersection between differential genes and disease-related genes. The STRING database (https://string-db.org/) analyzes protein–protein interactions encoded by genes. For visualizing the protein–protein interaction networks, Cytoscape 3.6.1 software is employed.

The CytoHubba plugin, based on Cytoscape 3.6.1 software, is also utilized to further filter core genes based on MCC values. The dynamic network Venn diagram could be created using the tools available on the Kidio bioinformatics cloud platform. We performed KEGG enrichment analysis on the differentially expressed genes obtained from transcriptome sequencing of Caco-2 cells using the KOBAS database. Genes with a P-value < 0.05 were considered statistically (Liu et al. 2018; Bu et al. 2021).

### Plasmid transfection and cell experiment grouping

Colorectal cancer cells in a healthy state were collected and digested using pancreatic enzymes. The cells were then seeded in a 24-well plate at a density of 8 × 10^3^ cells per well and cultured until they formed a monolayer. Subsequently, the culture medium should be removed, and the cells should be transfected using the Lipofectamine 3000 protocol (L3000150, Invitrogen, Thermo Fisher Scientific, Waltham, MA, USA). Following transfection, the cells were cultured at 37 °C with 5% CO2 for 6 to 8 h. After replacing the complete medium, the cells were further cultured for 48 h to allow extraction of RNA and protein for subsequent experiments.

The specific groups are as follows:

1. The oe-NC group and the oe-SIRT5 group;
2. The groups included the PBS group, LEVs group, LEVs + oe-NC group, and LEVs + oe-SIRT5 group. The LEVs were administered at a concentration of 1 μg and allowed to act for 24 h.
3. sh-NC group, sh-SIRT5#1 group, and sh-SIRT5#2 group;
4. sh-NC group, sh-p53#1 group, and sh-p53#2 group;
5. control group (PBS + sh-NC), LEVs + sh-NC group, LEVs + sh-p53 group.

Following a 48-h transfection period of colorectal cancer cells with plasmids, certain groups will receive a treatment of 1 μg of LEVs for 24 h. The optimal concentration for each plasmid should be determined by consulting the instruction manual and then adjusted based on the specific circumstances. The overexpression plasmid for SIRT5 (oe-SIRT5) and its control plasmid (oe-NC) was obtained from RayBiotech. The plasmid vector pEXP-RB-Mam (R11091.1, RayBiotech, Guangzhou, China) was used for construction. The following content contains the target sequences for sh-SIRT5#1 (TRCN0000018546) [CGTCCACACGAAACCAGATTT], sh-SIRT5#2 (TRCN0000232661) [AGAATTACAAGAGTCCAATTT], sh-p53#1 (TRCN0000003754) [TCAGACCTATGGAAACTACTT], sh-p53#2 (TRCN0000003753) [CGGCGCACAGAGGAAGAGAAT], and sh-NC [CTCGCTTGGGCGAGAGTAA] (Jin et al. 2017). Purchased from Sigma-Aldrich (USA) (Yang et al. 2020; Ye et al. 2020).

### EdU staining

Colorectal cancer cells exhibiting favorable growth conditions were cultured in a 12-well plate and transfected. After 48 h, perform EdU detection to analyze cell proliferation. The cells were incubated with the EdU reaction system (C0071S, BeyoClick™ EdU-488 Cell Proliferation Assay Kit, Beyotime, Shanghai, China) solution for 2 h. After that, the cells were fixed with 4% paraformaldehyde and washed three times with PBS. Subsequently, a single wash with 0.5% TritonX-100 was performed. Then, add DAPI solution to stain the cells. After three washes with PBS, images were captured from the fluorescence microscope for the subsequent calculation of the proliferation rate (Yao et al. 2022).

### Clone formation experiment

Colon cancer cells were collected from various groups and re-suspended following digestion with 0.25% trypsin. Inoculate each group of cells, consisting of 200 cells per well, into 10 mL of culture medium. Gently shake the mixture to ensure an even distribution of cells. In the transfection experiment, the fresh culture medium was replaced every two days after transfection, and this replacement occurred for 12 h. Re-transfect cells on day 6. The cells were fixed with a solution of more than 4% paraformaldehyde (P1110, Solarbio, Beijing, China) for 6 days and subsequently stained with a 0.1% crystal violet staining solution (G1063, Solarbio, Beijing, China) for 15 min. Cell colonies containing more than 50 cells should be counted utilizing a stereo microscope (Chen et al. 2020; Zhou and Zhao 2019; Xiang et al. 2019). The experiment is repeated three times.

### Flow cytometry

Colorectal cancer cells were cultured and transfected with corresponding plasmids for 48 h. Subsequently, the cells were transferred to collection tubes, fixed with pre-chilled 75% ethanol at 4 °C overnight. The cells were then washed thrice with ice-cold PBS and resuspended in 100 μL PBS. Following this, 0.5 mL of PI/RNase staining buffer (550,825, BD Biosciences, USA) was added, and the cells were incubated in the dark at room temperature for 15 min. Flow cytometry was performed within 24 h for PI staining. Finally, cell cycle distribution was determined using a flow cytometer (Guava® easyCyte™ 6-2L Base System, 0500–5007, Luminex, https://www.luminexcorp.com/) (Yao et al. 2022).

### Lactic acid production and glucose intake

The lactate production in tissues and cells was measured using the lactate assay kit (MAK064, Sigma-Aldrich, USA). Mix the cellular or tissue samples with a lactic acid analysis buffer at a four times greater volume for dissolution. Centrifuge the mixture at 13,000 g for 10 min. Remove the residue and eliminate LDH using a 10 KDa ultrafiltration centrifuge tube. Subsequently, transfer the resulting supernatant (50 μL) to a 96-well plate. Add 50 μL of lactic acid analysis buffer to each well. Thoroughly mix the contents using a pipette. Next, incubate the mixture at room temperature in a dark environment for 30 min. Finally, use a fluorescence plate reader with Ex/Em = 540/590 nm to monitor the changes in fluorescence intensity. Glucose uptake in tissues and cells was assessed using a fluorescent assay kit (MAK084, Sigma-Aldrich, USA) to determine glucose levels. Colon mucosal cells were isolated from clean-treated mouse intestines and cultured in a glucose-free RPMI medium. Subsequently, the cells were incubated for 60 min under either 1.0 mM or 20 mM glucose conditions to measure glucose uptake. Colon cancer cells were cultured in fresh low-glucose DMEM medium supplemented with 200 μM 2-NBDG. The cells were then incubated for 10 min under either 20 mM glucose or glucose-free conditions to measure glucose uptake (Fan et al. 2018; Lin et al. 2010).

### The Co-IP experiment detects protein–protein interactions

Transfect Flag-SIRT5 (plasmid 13,816, addgene, USA) or HA-p53 (plasmid D3033, Beyotime, Shanghai, China) plasmids into 293 T cells to detect exogenous proteins using co-immunoprecipitation (co-IP) assays. Additionally, conduct co-IP experiments to detect endogenous proteins in Caco-2 and SW480 cells.

Initially, the cells were lysed using IP lysis buffer (P0013, BiyunTian, Shanghai, China) enriched with protease and phosphatase inhibitors. The cell lysate was centrifuged at 12,000 g for 20 min at 4 °C. Next, the 293 T cell lysates, which contained 200 μg of protein, were treated with either an anti-Flag antibody (mouse; 1:50, F3165, Sigma-Aldrich, USA) or anti-HA antibody (mouse; 1:50, H9658, Sigma-Aldrich, USA) at 4℃ for 4 h. Either an anti-p53 antibody (mouse; 1:50, 60,283–2-Ig, Proteintech, Wuhan Sanying, China) or IgG (1:50, #3900, Cell Signaling, USA) was added to the lysates of Caco-2 and SW480 cells and incubated overnight at 4 °C. Next, protein A/G Sepharose beads (sc-2003, Santa Cruz, USA) were used to capture the antibody-protein complexes. After thoroughly washing, the complex was boiled in 1 × SDS sample buffer and subjected to Western blot analysis.

The antibodies used for Western blot analysis included rabbit anti-Flag antibody (1:1000, F7425, Sigma-Aldrich, USA), rabbit anti-HA antibody (1:1000, #3724, Cell Signaling, USA), rabbit anti-SIRT5 antibody (1:2000, 15,122–1-AP, Proteintech, Wuhan Sanying, China), and rabbit anti-p53 antibody (1:5000, 10,442–1-AP, Proteintech, Wuhan Sanying, China) (Zhang et al. 2019a, 2019b).

### *In vitro* succinylation experiment

Transfect 293 T cells with HA-p53 plasmid. The reaction system comprises an amber acylation buffer containing 20 mM HEPES at pH 8.0, 1 mM dithiothreitol, 1 mM phenylmethylsulfonyl fluoride, and 0.1 mg/mL bovine serum albumin. Different concentrations (0.1, 0.2, 0.4 mM) of amber acyl-CoA (S1129, Sigma-Aldrich, USA) were used. Incubate the reaction mixture at 30 °C for 15 min. The reaction was stopped by adding a loading buffer, and SDS-PAGE was performed. The succinylated p53 protein and other proteins were examined using co-IP and Western blot analysis. For this purpose, a rabbit pan-Succinyl-K antibody (#PTM-401, 1:1000 dilution, PTM Bio, Hangzhou, Zhejiang) was employed (Liu et al. 2022; Yang et al. 2018).

### In vitro desuccinylation experiment

The highly succinylated HA-p53 protein, purified from 293 T cells, was incubated with purified SIRT5 protein (ab101134, Abcam, UK) or Flag-SIRT5 (wild-type or enzymatically deficient mutant H158Y) for 1 h at 30 °C, with or without 1 mM NAD + . The incubation was carried out in succinate dehydrogenase buffer. The composition of the Amber enzyme buffer is as follows: 50 mM Tris–HCl (pH 8.0), 100 mM NaCl, 8 mM MgCl2, 20% glycerol, 1 mM DTT, 1 mM PMSF, and 0.1 mg/mL BSA. After adding the loading buffer, the reaction should be stopped, and then the separation could be carried out using SDS-PAGE. Analyze proteins through co-IP and Western blot. The deacetylation status of p53 was examined in different treatment groups, such as Caco-2 and SW480 cells, mouse colon polyp tissues, and colon cancer patient tissues, using co-immunoprecipitation (co-IP) and Western blot techniques (Liu et al. 2022; Yang et al. 2018).

### RT-qPCR

Total RNA was extracted from colon cancer cells using Trizol reagent, and the concentration and purity of the extracted RNA were measured using a Nanodrop 2000 microvolume UV–Vis spectrophotometer. The RNA was reverse transcribed to synthesize cDNA using the protocol specified in the PrimeScript RT reagent Kit (RR047A, Takara, Japan). The conditions for reverse transcription are as follows: incubate at 42℃ for 30 to 50 min, followed by heating at 85℃ for 5 s. qRT-PCR detection was done using the Fast SYBR Green PCR kit (RR820A, Takara, Japan) and the ABI PRISM 7300 RT-PCR system (Applied Biosystems). The reaction conditions were as follows: pre-denaturation at 95 °C for 5 min, denaturation at 95 °C for 30 s, annealing at 57 °C for 30 s, and extension at 72 °C for 30 s, with a total of 40 cycles. Each hole is set with 3 repetitions. β-Actin is utilized as an internal reference, and the 2^−ΔΔCt^ method is employed to assess the relative expression level of the gene. ΔΔCt is calculated as the difference between the average Ct value of the target gene in the experimental group and the average Ct value of the housekeeping gene in the experimental group, subtracted from the difference between the average Ct value of the target gene in the control group and the average Ct value of the housekeeping gene in the control group. The experiment was repeated 3 times (Jin et al. 2019). Table S1 provides the primer sequences' details.

### Western blot

Different groups of cells and tissues were collected and individually added to RIPA lysis buffer (P0013B, Beyotime, Shanghai, China) with 1% PMSF on ice for 30 min. The samples were centrifuged at 14,000 g and 4 ℃, and the supernatant was collected. The protein extraction solution was utilized to measure the protein concentration of the samples using the BCA method (P0012S, Bi Yun Tian, Shanghai, China). Add an appropriate 5 × loading buffer and boil at 100 °C for 10 min to denature the proteins. The protein loading amount is 50 μg. First, separate the gel for electrophoresis and then concentrate it. Next, transfer the bands that contain the target protein to a PVDF membrane after electrophoresis. The PVDF membrane was first immersed in a solution containing 5% skim milk powder and then sealed at room temperature for 1 h. Subsequently, the membrane was incubated overnight at 4 °C with primary antibodies, including anti-Flag (rabbit; dilution 1:1000, F7425, Sigma-Aldrich, USA), anti-HA (rabbit; dilution 1:1000, #3724, Cell Signaling, USA), anti-SIRT5 (rabbit; dilution 1:2000, 15,122–1-AP, Proteintech, Wuhan Sanying, China), anti-p53 (rabbit; dilution 1:5000, 10,442–1-AP, Proteintech, Wuhan Sanying, China), anti-pRb (rabbit; dilution 1:1000, #8516, Cell Signaling, USA), anti-Cyclin D1 (mouse; dilution 1:5000, 60,186–1-Ig, Proteintech, Wuhan Sanying, China), anti-p27 (rabbit; dilution 1:1000, 25,614–1-AP, Proteintech, Wuhan Sanying, China), anti-GLUT1 (rabbit; dilution 1:1000, #73,015, Cell Signaling, USA), anti-HKII (rabbit; dilution 1:2000, 22,029–1-AP, Proteintech, Wuhan Sanying, China), and anti-β-actin (rabbit; dilution 1:1000, ab8226, Abcam, UK). β-actin was used as the internal reference. Afterward, the membrane was washed with PBS-T at room temperature and subsequently incubated with secondary antibodies, which were HRP-labeled goat anti-rabbit/goat anti-mouse IgG (dilution 1:10,000, BA1054/BA1050, Boyade, Wuhan, China), at room temperature for 1 h. Finally, the membrane underwent 6 washes with PBS-T, each lasting 5 min. The ECL reaction solution (AR1172, BoDe, Wuhan, China) should be evenly applied onto the membrane and then subjected to exposure using the Amersham Imager 600 (USA). Then use Image J for grayscale analysis (Salem et al. 2019; Shu et al. 2011). The experiment is repeated three times.

### Construct a colitis-associated colon cancer (CAC) mouse model

Male p53 gene knockout mice (6 weeks old; p53-/-; on a C57BL6 background strain) were purchased from Saibaio (C001203). Wild-type (WT) male C57BL6 mice, aged 6 weeks, were obtained from Vital River, Beijing, China. The animals were kept in SPF-level animal rooms with a constant humidity of 45%-50% and a temperature range of 25 ~ 27 ℃ for one week. Each day, they were exposed to a 12-h light and 12-h dark cycle to adapt to the experimental environment.

The wild-type (WT) and p53 knockout (p53-/-) mice were assigned to four groups, namely the WT group, p53-/- group, LEVs + WT group, and LEVs + p53-/- group, each consisting of 6 mice. To establish the CAC model, wild-type (WT) and p53-/- mice were intraperitoneally injected with a single dose of azoxymethane (AOM) at 10 mg/kg (Sigma-Aldrich, St Louis, MO, A5486). After five days, a 2.5% solution of dextran sulfate sodium (DSS) was injected into the drinking water for five days. It was followed by 14 days of regular drinking water. After the last administration of 2.5% DSS, 3 weeks elapsed, and then 100 μg (100 μL PBS) of LEVs or PBS (as the control) were injected twice a week via the tail vein. The mice were euthanized 69 days after the AOM injection. Subsequently, the colon mucosa and polyp tissues were dissected for Western blot and immunohistochemistry analysis. Polyp counting and immunohistochemical analysis were conducted by researchers blinded to the treatment methods. The number of polyps is determined by counting the total polyps in a specific mouse. Polyp burden in mice is determined by calculating the sum of the diameters of all polyps (Mao et al. 2022; Neufert et al. 2007).

### Immunohistochemistry

The expression of proliferation-related markers Ki67, Cyclin D1, and p27 was detected in mouse colon polyp tissue using the streptavidin peroxidase (SP) immunohistochemical staining method. Moreover, the expression of SIRT5 was examined in both colorectal cancer tissue and adjacent normal tissue of colorectal cancer patients. Colonic polyp tissue and colon cancer tissue paraffin specimens were obtained from patients. After routine dehydration processing, consecutive sections with a thickness of 5 μm were performed. Subsequently, the specimens were stained using the conventional procedure of immunohistochemistry. To block endogenous peroxidases, the samples were treated with 3% hydrogen peroxide at room temperature for 10 min and then subjected to a 10-min blocking step using normal goat serum. The primary antibodies used were rabbit anti-Ki67 (1:2000, 28,074–1-AP, Proteintech, Wuhan, China), mouse anti-Cyclin D1 (1:500, 60,186–1-Ig, Proteintech, Wuhan, China), rabbit anti-p27 (1:200, 25,614–1-AP, Proteintech, Wuhan, China), and rabbit anti-SIRT5 (1:200, 15,122–1-AP, Proteintech, Wuhan, China). The incubation was carried out overnight at 4 ºC. Following this, the tissue sections were incubated with biotinylated secondary antibodies (Sheep anti-Mouse/Sheep anti-Rabbit, diluted 1:500, BA1001/BA1003, Boster, Wuhan, China) at 37 ºC for 20 min. Subsequently, 50μL of streptavidin-peroxidase solution was added and incubated at room temperature for 10 min. Stain the slides using DAB, followed by restaining with hematoxylin. Finally, dehydrate, clear, and mount the slides for microscopic observation. Using an isotype control instead of PBS is recommended as a negative control.

The criteria used to determine protein-positive cells are as follows: standard positive cells display a brownish-yellow color, and scoring is based on the extent and intensity of staining. The percentage of positively stained area assesses the staining range: score 0 signifies < 5%, score 1 signifies 5–25%, score 2 signifies 25–50%, score 3 signifies 50–75%, and score 4 signifies > 75%. The staining intensity is classified into four levels: 0, 1, 2, and 3. Each number corresponds to a specific staining intensity: 0 represents negative (no staining), 1 represents mild (weak) staining, 2 represents moderate staining, and 3 represents intense (strong) staining. Next, the staining intensity should be multiplied by the percentage of area to calculate the weighted score (Li et al. 2016; Tian and Yuan 2018).

### Patient and sample preparation

A total of 58 fresh colon cancer tissues and their matched adjacent non-tumor tissues were collected from colon cancer patients who underwent curative colon resection surgery at our hospital between 2017 and 2018, without receiving any chemotherapy or radiotherapy. Among them, there were 33 male and 25 female patients, with a mean age of 55.97 ± 9.24. Nineteen cases had lymph node invasion, while 39 cases did not; 39 cases were classified as TNM stage I-II, and 19 cases as stage III. The 58 colon cancer patient tissues were divided into high and low expression groups based on the median expression levels of SIRT5 and p53 succinylation proteins. Survival analysis was conducted on patients with high and low expression of SIRT5 and p53 succinylation proteins, with a follow-up period of up to 5 years. (Tian and Yuan 2018).

## Statistical analysis

SPSS (version 21.0, IBM, USA) was utilized as the statistical analysis software in this study. Measurement data is typically represented by the mean value plus or minus the standard deviation. Initially, perform tests to assess normality and homogeneity of variance to confirm the fulfillment of the assumptions of normal distribution and homogeneity of variance. Between-group comparisons were conducted using independent t-tests, while multiple-group comparisons were analyzed using one-way analysis of variance (ANOVA) or repeated measures analysis of variance, followed by post hoc comparisons (Tukey's). The survival time of patients with high/low expression of SIRT5 and p53 succinylation proteins is compared using a log-rank test. A p-value less than 0.05 indicates that the observed difference is statistical (Velazquez et al. 2019; Wang et al. 2023; Tian and Yuan 2018).

## Results

### Exploring the regulatory effects of *L. plantarum*-derived extracellular vesicles on colorectal cancer progression through *in vitro* studies and bioinformatics analysis

Probiotics are crucial in regulating the microbiota and maintaining gut homeostasis. For instance, *L. plantarum* has been shown to enhance immune function in the intestinal mucosa and improve the integrity of the skin barrier (Hao et al. 2021; Kim et al. 2020). Furthermore, several studies have demonstrated the inhibitory effects of *L. plantarum* on the proliferation and metastasis of colon cancer cells in mice. Additionally, it has been suggested that LEVs could mitigate DSS-induced ulcerative colitis through modulation of the gut microbiota (Yue et al. 2020a, b; Hao et al. 2021).

To explore the potential regulatory effects of LEVs on the progression of colorectal cancer, we initially conducted an *in vitro* culture of *L. plantarum*. A 5% inoculum of L.plantarum was added to MRS broth and incubated for 0–48 h. The results of the bacterial colony count are presented in Figure S1A. After 24 h of cultivation, there was a drop in the extracellular pH from 6.56 to 3.70 (Figure S1B). This drop in pH indicates that 24 h is the ideal duration for cultivating bacteria and producing biomass. Thus, the cultivation duration for *L. plantarum* was determined to be 24 h.

**Fig. 1:**
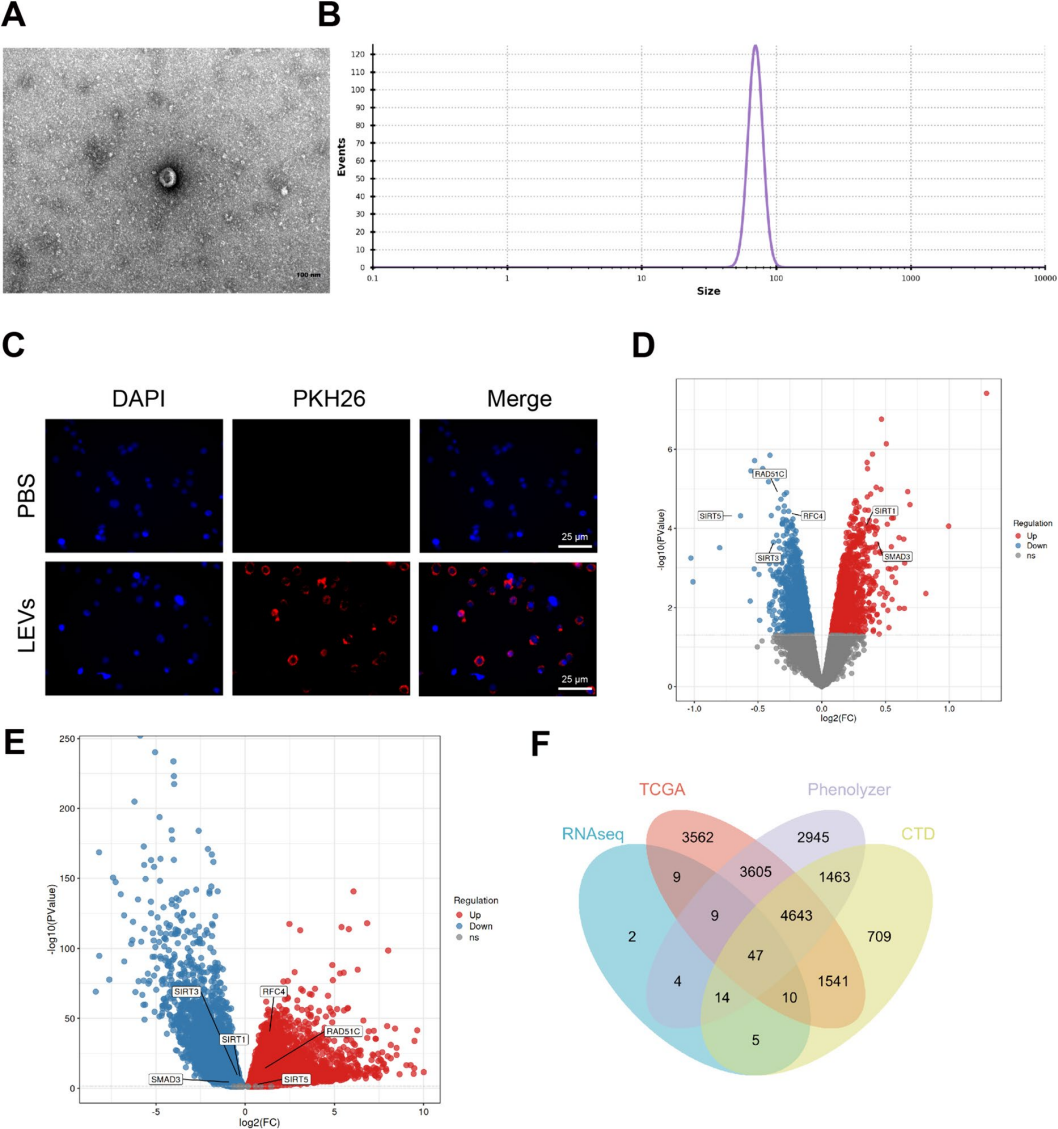
Key Genes Regulating Colorectal Cancer Progression Selected through Bioinformatics Analysis of LEVs. Note: (**A**) Morphological characteristics of LEVs analyzed by transmission electron microscopy (scale bar = 50 nm); (**B**) Particle size of LEVs measured by NTA; (**C**) Immunofluorescence detection of LEVs uptake by Caco-2 cells (scale bar = 25 μm), DAPI (blue) staining represents cell nuclei, PKH26 (red) represents LEVs; (**D**) Volcano plot showing differentially expressed genes in Caco-2 cells treated with and without LEVs, with each group consisting of four samples; (**E**) Volcano plot showing differentially expressed genes in TCGA-COAD high-throughput sequencing dataset, Normal group, n = 41, Tumor group, n = 483; (F) Venn diagram showing the intersection of differentially expressed genes in RNAseq and TCGA-COAD datasets with colorectal cancer-related genes in Phenolyzer and CTD databases

LEVs were isolated and extracted from *L. plantarum*. Transmission electron microscopy confirmed that LEVs exhibited the characteristic cup-shaped vesicular morphology (Fig. 1A). NTA analysis demonstrated that most LEVs had an average diameter of 72.51 ± 4.96 nm (Fig. 1B). LEVs were labeled with PKH26 and co-cultured with Caco-2 cells. The uptake of LEVs by Caco-2 cells was observed using a fluorescence microscope. The results revealed that PKH26-labeled LEVs displayed red fluorescence in Caco-2 cells (Fig. 1C), indicating the internalization of LEVs by Caco-2 cells.

We collected Caco-2 cell samples, both untreated and treated with LEVs, for high-throughput sequencing analysis. The volcano plot (Fig. 1D) presents the differential analysis results, revealing 2607 differentially expressed genes. Among them, 1390 genes were upregulated, while 1217 genes were downregulated. We performed differential analysis on the transcriptome sequencing dataset of colorectal cancer obtained from the TCGA database. This analysis revealed differential expression in 13,426 genes, with 7,999 genes upregulated and 5,427 genes downregulated (Fig. 1E). The differentially expressed genes in Fig. 1D and 1E intersected with the colon cancer-related genes in the database, resulting in 47 intersecting genes (Fig. 1F).

The above findings indicate that we have successfully identified 47 key genes implicated in regulating colorectal cancer progression using bioinformatics analyses.

### Unraveling the regulatory role of *L. plantarum*-derived extracellular vesicles in colorectal cancer progression: a focus on sirt5 and related gene networks

**Fig. 2:**
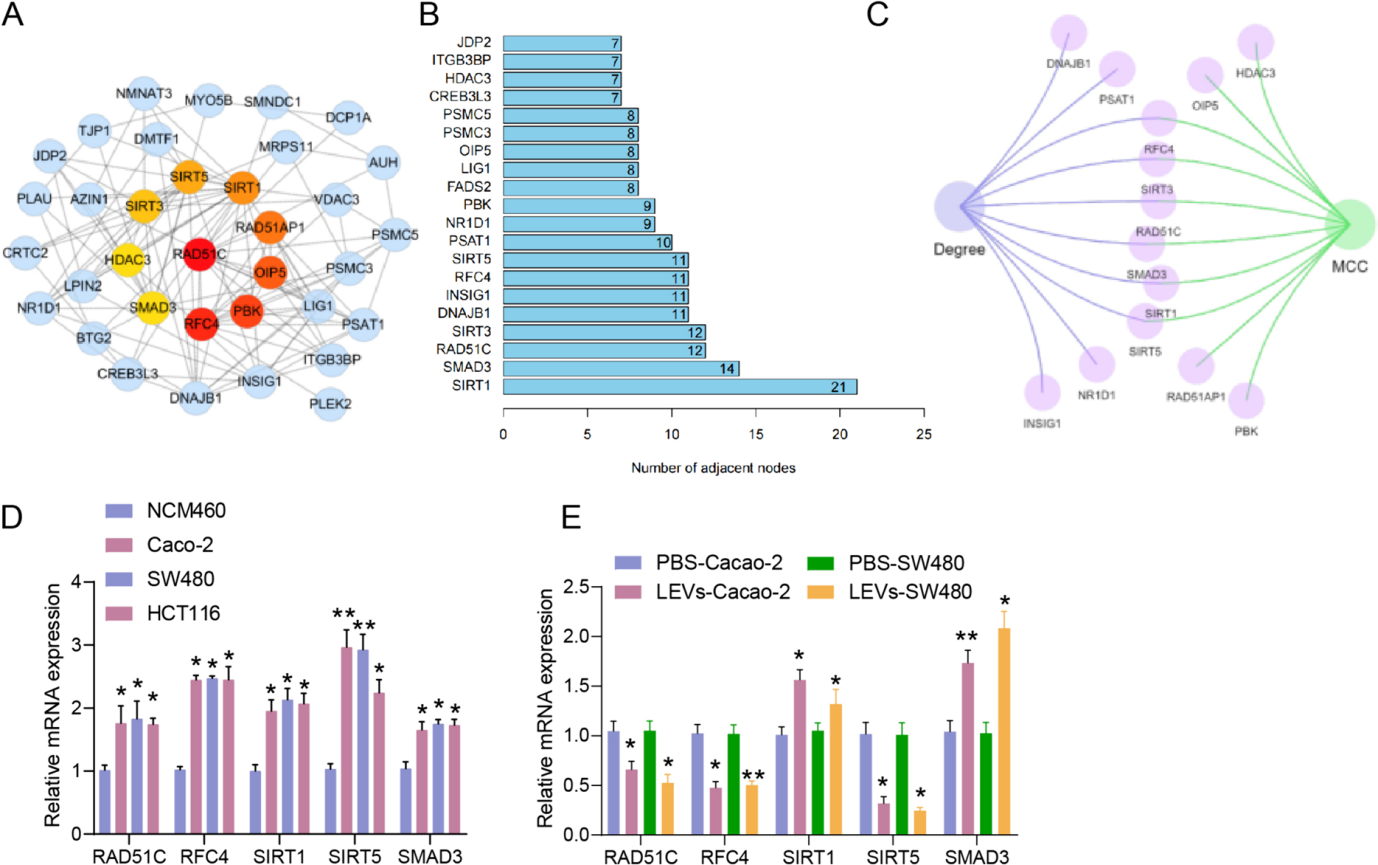
Target Genes Regulating Colorectal Cancer Progression Selected through Bioinformatics Analysis of LEVs—SIRT5. Note: (**A**) The interactive network of the top 10 core proteins encoded by the 47 intersecting genes following MCC sorting, marked in red in the right panel; (**B**) Bar graph showing the Degree ranking of proteins encoded by the 47 intersecting genes, with the top 20 proteins displayed; (**C**) Venn diagram showing the intersection network of Degree and top 10 proteins ranked by MCC; (**D**) RT-qPCR detection of mRNA expression of RAD51C, RFC4, SIRT1, SIRT5, and SMAD3 in human normal colonic mucosal cells and various colorectal cancer cell lines, * indicates P < 0.05 compared to NCM460 cells, ** indicates P < 0.01 compared to NCM460 cells; (**E**) RT-qPCR detection of mRNA expression of RAD51C, RFC4, SIRT1, SIRT5, and SMAD3 in Caco-2 and SW480 cells treated with LEVs, * indicates P < 0.05 compared to PBS group, ** indicates P < 0.01 compared to PBS group. Cell experiments were repeated three times

To identify additional target genes involved in regulating colorectal cancer progression by LEVs, the protein encoded by the earlier obtained set of 47 intersecting genes was imported into the STRING database to analyze protein–protein interaction networks (Fig. 2A). The core proteins were then selected using the CytoHubba plugin of Cytoscape 3.6.1 software and sorted based on the MCC value. The top 10 proteins, in order, are RAD51C, RFC4, PBK, OIP5, RAD51AP1, SIRT1, SIRT5, SIRT3, HDAC3, and SMAD3. Additionally, sorting was conducted based on the Degree value (Fig. 2B). The intersection of Degree and the top 10 MCC proteins resulted in RAD51C, RFC4, SIRT1, SIRT5, SIRT3, and SMAD3 (Fig. 2C). RAD51C, RFC4, and SIRT5 exhibited a low differential expression in Caco-2 cells treated with LEVs but a high differential expression in TCGA colon cancer tissue samples. Conversely, SIRT1 and SMAD3 displayed high differential expression in LEV-treated Caco-2 cells while showing low differential expression in TCGA colon cancer tissue samples. SIRT3 consistently showed low expression in all cases, as demonstrated in Fig. 1D-E volcano plot.

Next, we confirmed the expression of RAD51C, RFC4, SIRT1, SIRT5, and SMAD3 genes in different colon cancer cell lines. The RT-qPCR results demonstrated that the expression of multiple genes in human colon cancer cell lines, namely Caco-2, SW480, and HCT116, was higher compared to the normal human colonic mucosal cell line NCM460. Notably, Caco-2 and SW480 cells exhibited the most substantial change in gene expression, specifically in the case of SIRT5 (Fig. 2D). Subsequent investigation unveiled underexpression of RAD51C, RFC4, and SIRT5 in Caco-2 and SW480 cells treated with LEVs, while SIRT1 and SMAD3 were considerably overexpressed. Notably, SIRT5 displayed the most pronounced expression alteration (Fig. 2E).

Based on the results above, we hypothesize that SIRT5 could serve as a crucial target gene in regulating colon cancer progression by LEVs.

### Impact of *L. plantarum*-derived extracellular vesicles on colorectal cancer cell proliferation and metabolism: the modulatory role of SIRT5

**Fig. 3:**
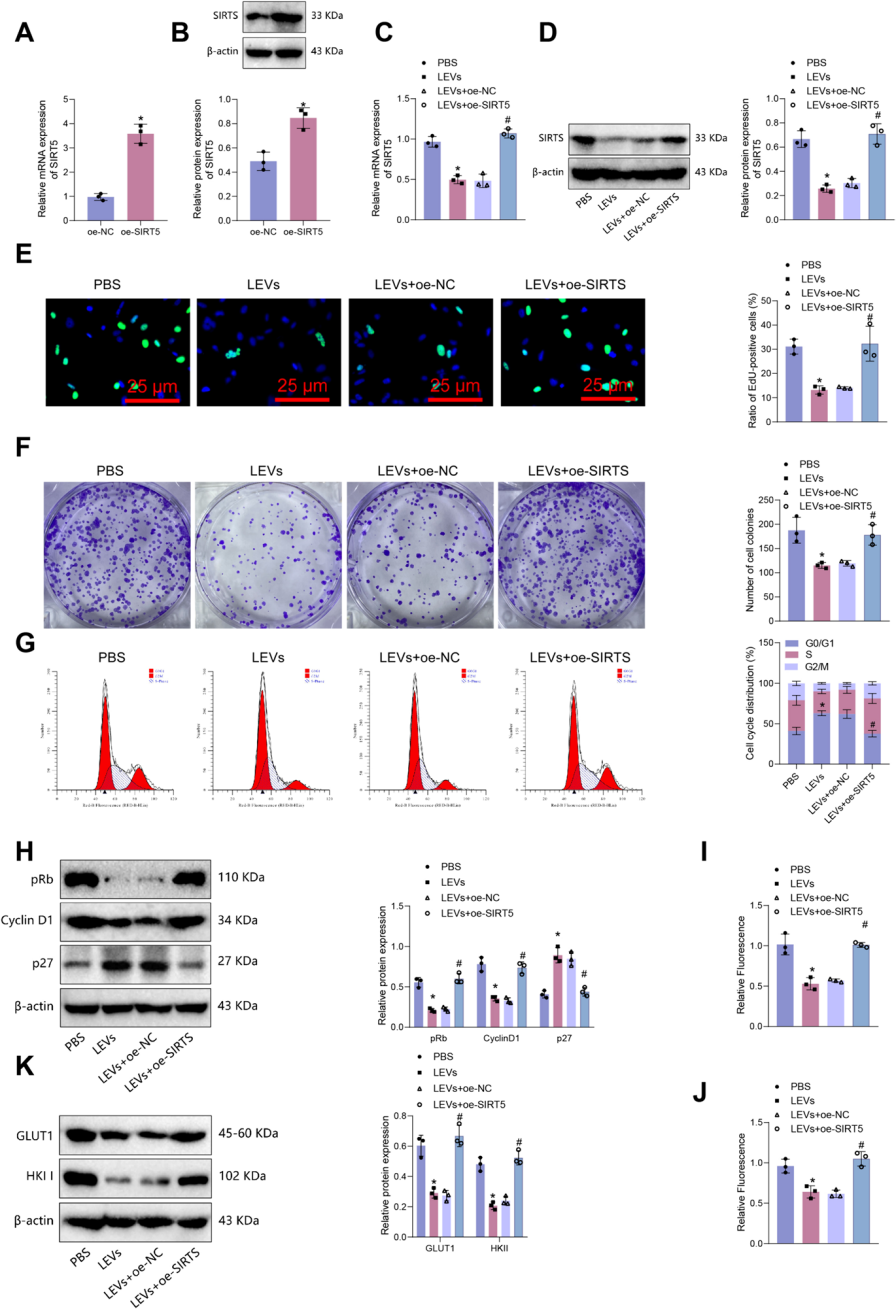
The Effect of LEVs-Mediated SIRT5 Expression Regulation on Proliferation and Glycolysis in Caco-2 Colorectal Cancer Cells. Note: (**A**-**B**) RT-qPCR and Western blot detection of SIRT5 mRNA and protein expression in Caco-2 cells overexpressing SIRT5; (**C**-**D**) RT-qPCR and Western blot detection of SIRT5 mRNA and protein expression in different groups of Caco-2 cells; (**E**) EdU staining to detect proliferation of different groups of Caco-2 cells (scale bar = 25 μm); (**F**) Clonogenic assay to detect colony formation of different groups of Caco-2 cells; (**G**) Flow cytometry analysis to detect cell cycle changes in different groups of Caco-2 cells; (**H**) Western blot detection of expression changes of cell cycle-related proteins in different groups of Caco-2 cells; (**I**) Glucose uptake in different groups of Caco-2 cells; (**J**) Lactate generation in different groups of Caco-2 cells; (**K**) Western blot detection of expression of glycolysis rate-limiting enzymes in different groups of Caco-2 cells. * indicates P < 0.05 compared to oe-NC or PBS group, # indicates P < 0.05 compared to LEVs + oe-NC group. Cell experiments were repeated three times

Research reports indicate that the probiotic strain B282 of *Lactobacillus plantarum* effectively inhibits the growth of human colon cancer cells (Caco-2) in a time and dose-dependent manner, demonstrating clear anti-proliferative activity (Saxami et al. 2017). Therefore, we conducted a further investigation to determine if LEVs regulate the expression of SIRT5 and affect the proliferation of colon cancer cells. SIRT5 was initially overexpressed in Caco-2 and SW480 cells. RT-qPCR and Western blot analysis indicated an increase in SIRT5 expression in the oe-SIRT5 group compared to the oe-NC group (Fig. 3A-B; Figure S2A-B). Subsequent treatment with LEVs in Caco-2 and SW480 cells decreased SIRT5 expression compared to the PBS group. Moreover, the LEVs + oe-SIRT5 group demonstrated a notable increase in SIRT5 expression in the cells compared to the LEVs + oe-NC group (Fig. 3C-D; Figure S2C-D).

To evaluate cell proliferation, we performed EdU and clone formation experiments. The results revealed a noteworthy decrease in cell proliferation in the LEV group compared to the PBS group. Additionally, the LEVs + oe-SIRT5 group exhibited increased cell proliferation compared to the LEVs + oe-NC group (Fig. 3E-F; Figure S2E-F). The cell cycle plays a crucial role in regulating normal cell proliferation; however, it frequently experiences dysregulated control in tumor cells (Shen et al. 2020). We conducted additional analysis to investigate changes in the cell cycle. The analysis using flow cytometry showed an arrest of cells in the G0/G1 phase after LEVs treatment. However, the overexpression of SIRT5 caused a notable decrease in the proportion of cells in the G0/G1 phase after LEVs treatment (Fig. 3G; Figure S2G). Changes in the expression of cell cycle-related proteins were observed following treatment with LEVs. Specifically, the expression of CyclinD1 and pRb proteins in cells decreased, while the expression of p27 protein increased. Conversely, overexpression of SIRT5 considerably increased CyclinD1 and pRb protein expression and decreased p27 protein expression in LEVs-treated cells (Fig. 3H; Figure S2H).

The division and proliferation of tumor cells necessitate the uptake of glucose and other carbon sources over their energy requirements, leading to aberrant energy and synthetic metabolism in most tumor cells. Aerobic glycolysis is a key characteristic of the development and progression of cancer (Bauer et al. 2004; DeBerardinis et al. 2008; Moreno-Sánchez et al. 2007). Therefore, we also assessed the cellular glycolysis level. In the LEVs group, glucose uptake and lactate production were lower than in the PBS group. Additionally, glucose transporter protein GLUT1 and hexokinase II (HKII) expression was reduced. Conversely, in the LEVs + oe-SIRT5 group, both glucose uptake and lactate production showed an increase compared to the LEVs + oe-NC group, accompanied by an elevation in the expression of GLUT1 and HKII (Fig. 3I-K; Figure S2I-K).

The results above indicate that LEVs can potentially impede the proliferation of colon cancer cells and alter glycolysis metabolism through downregulating SIRT5 expression.

### Elucidating the mechanisms of SIRT5 in regulating colorectal cancer progression: interactions with p53 and modulation of succinylation

To investigate the downstream mechanisms by which SIRT5 regulates the proliferation and glycolysis of colon cancer cells, we conducted a pathway enrichment analysis on differentially expressed genes obtained from LEV-treated Caco-2 cells. The analysis revealed that these genes were predominantly enriched in several pathways, including the CCKR signaling map, EGF receptor signaling pathway, Apoptosis signaling pathway, PDGF signaling pathway, FGF signaling pathway, and p53 pathway (Figure S3A). Research reports suggest that p53 exhibits tumor-suppressive activity and plays a role in regulating the aerobic glycolysis pathway. Moreover, post-translational modifications (PTMs) are widely recognized as the most effective means of regulating p53 activation (Liu and Gu 2022). The protein p53 undergoes succinylation, a process that involves the addition of succinyl group to specific lysine residues, altering the conformation, stability, and interactions of p53 with other proteins. These modifications can regulate p53's DNA binding ability, modulate gene expression, induce apoptosis, thereby impacting its role in preventing cancer progression (Xu 2003). Moreover, SIRT5, a member of the sirtuin family, has been shown to have efficient desuccinylating activity on lysine residues, which regulates p53 desuccinylation (Liu et al. 2022). Protein interaction analysis was conducted on the candidate target genes that were previously identified, revealing an interaction between SIRT5 and the p53 protein (Figure S3B).

**Fig. 4:**
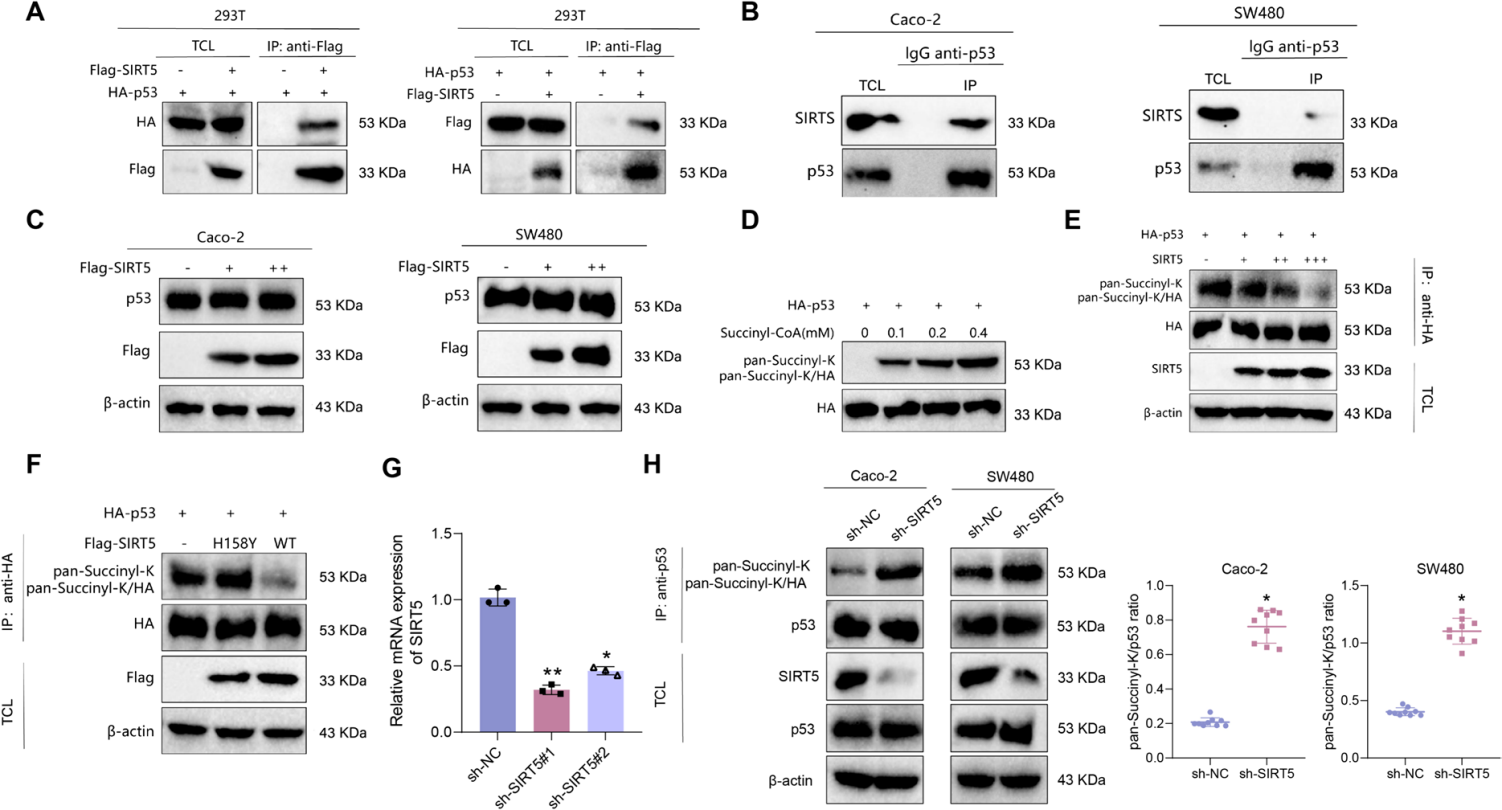
SIRT5-Mediated Depsuccinylation of p53. Note: (**A**) Co-IP experiment to detect the interaction between exogenous SIRT5 and p53 in 293 T cells; (**B**) Co-IP experiment to detect the interaction between endogenous SIRT5 and p53 in Caco-2 and SW480 cells; (**C**) Western blot detection of SIRT5 and p53 protein expression in Caco-2 and SW480 cells overexpressing SIRT5 (Flag-SIRT5); (**D**) Western blot detection of succinylation levels of p53 in 293 T cells after succinyl-CoA treatment; (**E**) Co-IP experiment to detect the desuccinylation effect of overexpressed SIRT5 on p53 in 293 T cells; (**F**) Co-IP experiment to detect the effect of co-transfection of HA-p53 with Flag-SIRT5 or its enzymatically deficient mutant H158Y on p53 succinylation levels in 293 T cells; (**G**) RT-qPCR detection of SIRT5 knockdown efficiency, * indicates P < 0.05 compared to sh-NC group, ** indicates P < 0.01 compared to sh-NC group; (**H**) Co-IP experiment to detect the effect of SIRT5 knockdown on p53 succinylation levels in Caco-2 and SW480 cells. TCL: total cell lysate; IP: immunoprecipitation. Cell experiments were repeated three times

To validate the interaction between SIRT5 and p53, we conducted co-immunoprecipitation (co-IP) experiments. The results demonstrated that in 293 T cells co-transfected with Flag-SIRT5 and HA-p53, Flag-SIRT5 effectively pulled down HA-p53 and vice versa, indicating a physical interaction between exogenous SIRT5 and p53 (Fig. 4A). Moreover, in Caco-2 and SW480 cells, endogenous p53 successfully pulled down endogenous SIRT5, further supporting a protein interaction between endogenous SIRT5 and p53 (Fig. 4B). Furthermore, Western blot analysis demonstrated that the overexpression of SIRT5 did not affect the protein levels of p53 in Caco-2 and SW480 cells (Fig. 4C).

To further investigate the impact of SIRT5 on p53 activity through desuccinylation modification, we conducted *in vitro* experiments to study succinylation and desuccinylation. Initially, HA-p53 was transfected into 293 T cells, and varying doses of Coenzyme A were introduced to induce acetylation. It was observed that the acetylation level of p53 was dependent on the dose administered (Fig. 4D). HA-p53 was transfected into 293 T cells and incubated with purified SIRT5 protein. The findings revealed that SIRT5 could deacetylate p53 in a dose-dependent manner, as shown in Fig. 4E. We then generated an enzymatically-deficient mutant of SIRT5, namely SIRT5-H158Y. Co-immunoprecipitation experiments demonstrated that the flawed SIRT5 variant, SIRT5-H158Y, could not remove succinyl groups from p53 (Fig. 4F). Finally, we investigated the influence of SIRT5 knockdown on the levels of p53 succinylation. The results of the knockdown efficiency tests are displayed in Fig. 4G. We chose sh-SIRT5#1 due to its superior knockdown efficiency for subsequent experiments. The results of Co-IP detection demonstrated an increase in the acetylation level of p53 in Caco-2 and SW480 cells following the knockdown of SIRT5 (Fig. 4H).

These results suggest that SIRT5 is capable of mediating the succinylation modification of p53.

### Deciphering the regulatory role of LEVs on colon cancer cell proliferation and glycolysis: involvement of the SIRT5/p53 Axis

**Fig. 5:**
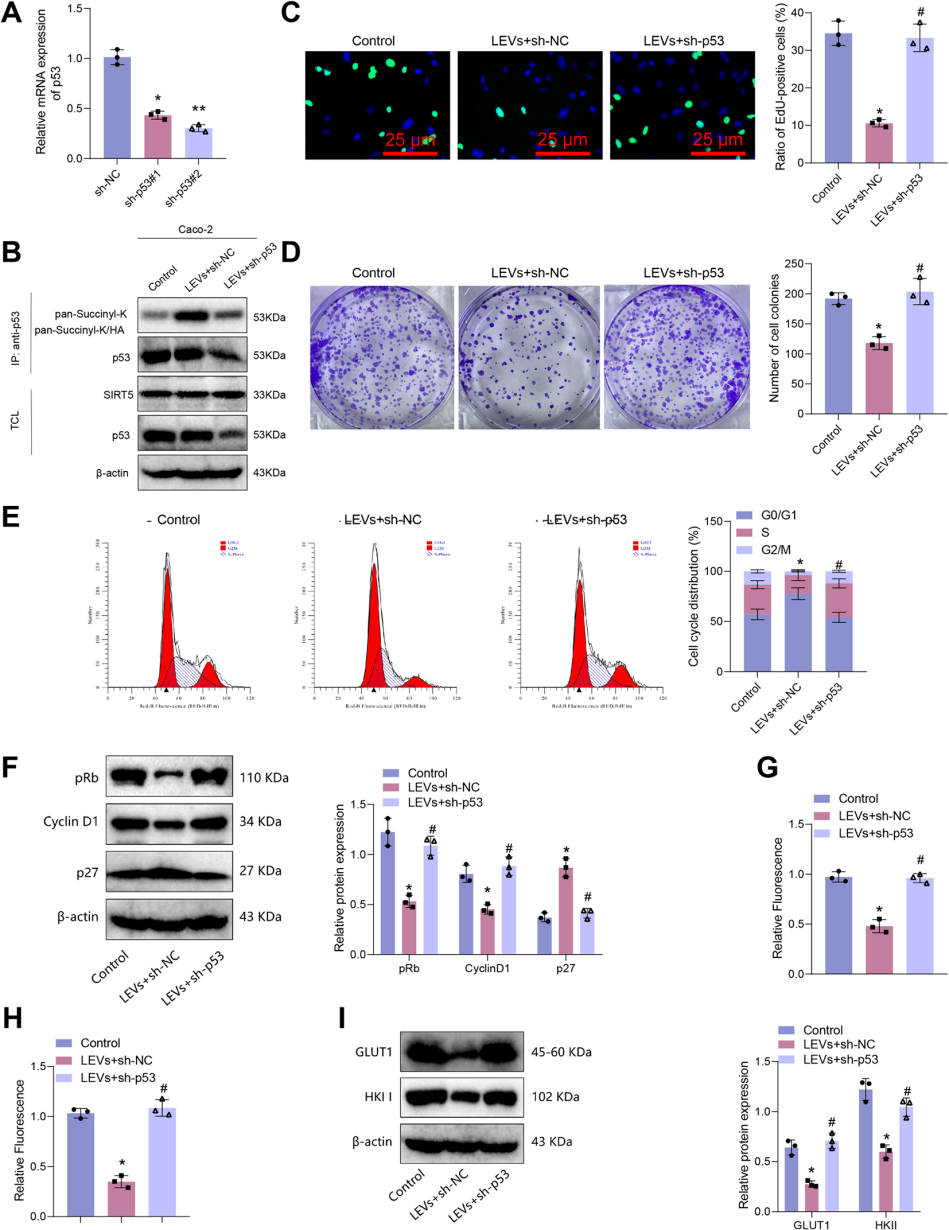
The Effect of LEVs-Mediated SIRT5/p53 Axis on Proliferation and Glycolysis in Caco-2 Colorectal Cancer Cells. Note: (**A**) RT-qPCR detection of p53 knockdown efficiency; (**B**) Co-IP experiment to detect succinylation levels of p53 in different groups of Caco-2 cells; (**C**) EdU staining to detect proliferation of different groups of Caco-2 cells (scale bar = 25 μm); (**D**) Clonogenic assay to detect colony formation of different groups of Caco-2 cells; (**E**) Flow cytometry analysis to detect cell cycle changes in different groups of Caco-2 cells; (**F**) Western blot detection of expression changes of cell cycle-related proteins in different groups of Caco-2 cells; (**G**) Glucose uptake in different groups of Caco-2 cells; (**H**) Lactate generation in different groups of Caco-2 cells; (I) Western blot detection of expression of glycolysis rate-limiting enzymes in different groups of Caco-2 cells. * indicates P < 0.05 compared to sh-NC or Control group, ** indicates P < 0.01 compared to sh-NC group, # indicates P < 0.05 compared to LEVs + sh-NC group. Cell experiments were repeated three times

Further investigation into the regulation of the SIRT5/p53 axis by LEVs on the proliferation and glycolytic metabolism of colon cancer cells revealed a study indicating an association between GLU-1 expression levels and glucose metabolism (Meng et al. 2019). The results of p53 knockdown efficiency testing, as shown in Fig. 5A, led to the selection of sh-p53#2 with better knockdown efficiency for subsequent experiments. The co-immunoprecipitation (co-IP) results revealed that the acetylation level of p53 was increased in the LEVs + sh-NC group compared to the Control group, whereas the expression of SIRT5 protein was reduced. Moreover, when comparing the LEVs + sh-NC group and the LEVs + sh-p53 group, both the acetylation level and protein level of p53 were decreased in the latter, while no changes were observed in the expression of SIRT5 protein (Fig. 5B; Figure S4A).

Cell proliferation analysis demonstrated a decrease in the proliferation of cells in the LEVs + sh-NC group compared to the Control group. Conversely, the proliferation of cells in the LEVs + sh-p53 group was increased compared to the LEVs + sh-NC group (Fig. 5C-D; Figure S4B-C). Analysis of cell cycle-related changes showed that the LEVs + sh-NC group exhibited an arrest in the G0/G1 phase compared to the Control group. Additionally, the LEVs + sh-p53 group showed a reduction in the proportion of cells in the G0/G1 phase compared to the LEVs + sh-NC group (Fig. 5E; Figure S4D). Western blot analysis revealed that the expression of CyclinD1 and pRb proteins was decreased in the LEVs + sh-NC group compared to the Control group, while the expression of p27 protein was increased. In contrast, the expression of CyclinD1 and pRb proteins was increased in the LEVs + sh-p53 group compared to the LEVs + sh-NC group, while the expression of p27 protein was decreased (Fig. 5F; Figure S4E).

Cellular glycolysis metabolism was assessed, and the findings demonstrated a reduction in glucose uptake and lactate production in the LEVs + sh-NC group compared to the Control group. The expression of glucose transporter 1 (GLUT1) and hexokinase II (HKII) also decreased. Conversely, the LEVs + sh-p53 group exhibited an increase in both glucose uptake and lactate production compared to the LEVs + sh-NC group, accompanied by upregulation of GLUT1 and HKII expression (Fig. 5G-I; Figure S4F-I).

The results above suggest that LEVs can regulate the SIRT5/p53 axis, thereby suppressing the proliferation of colon cancer cells and glycolysis.

### Unraveling the impact of *L. plantarum*-derived extracellular vesicles on colorectal tumor suppression: insights from a colitis-associated tumor model and the SIRT5/p53 Pathway

**Fig. 6:**
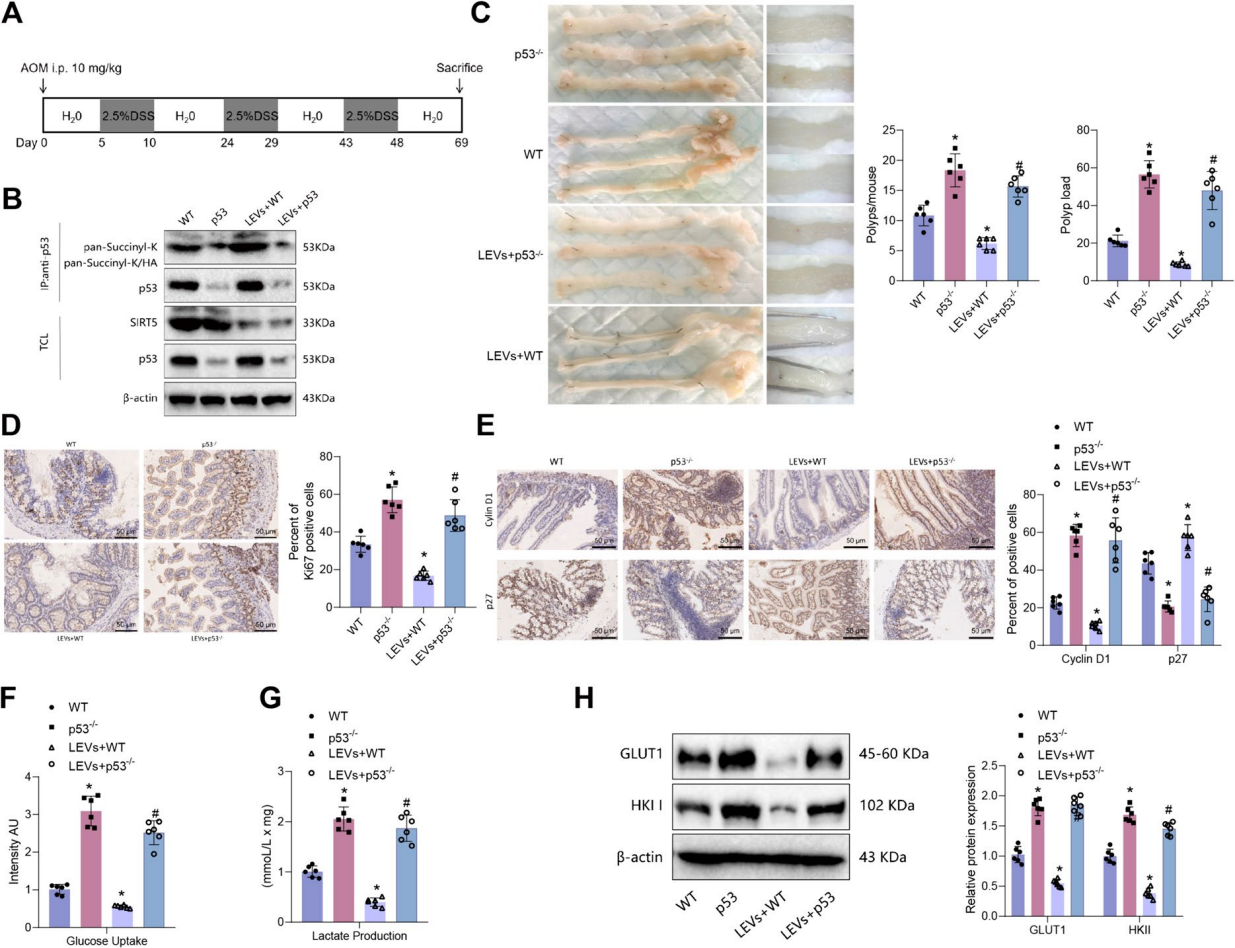
LEV-mediated regulation of the SIRT5/p53 axis affects proliferation and glycolysis in intestinal epithelial cells, influencing colon tumor formation. Note: (**A**) Schematic diagram illustrating the construction of a mouse model of colitis-associated tumors; (**B**) co-immunoprecipitation (co-IP) experiment detecting the acetylation level of p53 in mouse colonic polyp tissues from each group; (**C**) statistical analysis of the number and burden of colonic polyps in each group of mice; (**D**) immunohistochemical staining detecting the positive expression of Ki67 protein in mouse colonic polyp tissues from each group (scale bar = 100 μm); (**E**) immunohistochemical staining detecting the positive expression of cell cycle-related proteins Cyclin D1 and p27 in mouse colonic polyp tissues from each group (scale bar = 100 μm); (**F**) glucose uptake in mouse colonic polyp tissues from each group; (**G**) lactate production in mouse colonic mucosal tissues from each group; (**H**) Western blot detecting the expression of glycolytic rate-limiting enzymes GLUT1 and HKII in mouse colonic mucosal tissues from each group. * represents a difference compared to the WT group (P < 0.05), # represents a difference compared to the LEVs + WT group (P < 0.05), 6 mice per group

To further explore the occurrence of intestinal epithelial cell proliferation and glycolysis in the formation of colorectal tumors, we created a colitis-associated tumor model by using p53 knockout mice. The specific modeling process is depicted in Fig. 6A. Colon polyp tissues were collected for co-immunoprecipitation (co-IP) experiments. The results revealed an increase in the succinylation level of p53 in the LEVs + WT group compared to the WT group, accompanied by a decrease in the expression of SIRT5 protein. In contrast, both the succinylation level and protein expression of p53 were reduced in the p53-/- group or the LEVs + p53-/- group relative to the WT group or the LEVs + WT group. Interestingly, there was no noticeable change in the expression of SIRT5 protein (Fig. 6B).

Following treatment with AOM/DSS at day 69, the LEVs + WT group of mice exhibited a decrease in the number and size of colonic polyps compared to the WT group. Conversely, the p53-/- group or LEVs + p53-/- group of mice showed an increase in the number and size of colonic polyps compared to the WT or LEVs + WT group (Fig. 6C). Immunohistochemistry was used to detect the expression of cell proliferation and cycle-related proteins. The results revealed a decrease in Ki67 and Cyclin D1 protein expression in colonic polyp tissues of the LEVs + WT group compared to the WT group. Conversely, there was an increase in the expression of p27 protein. Furthermore, compared to the WT group or the LEVs + WT group, the colonic polyp tissues of the p53-/- group or the LEVs + p53-/- group exhibited an increase in the expression of Ki67 and Cyclin D1 proteins, accompanied by a decrease in the expression of p27 protein (Fig. 6D-E).

The study examined the level of glycolytic metabolism in colonic mucosal tissue. The findings demonstrated that the colonic mucosal tissue of the LEVs + WT group exhibited reduced glucose uptake and lactate production compared to the WT group. Moreover, the expression of glucose transporter protein GLUT1 and hexokinase II (HKII) exhibited a substantial decrease. In contrast, the colonic mucosal tissue of the p53-/- group or LEVs + p53-/- group exhibited increased glucose uptake and lactate production when compared to the WT group or the LEVs + WT group. Additionally, there was an increase in the expression of GLUT1 and HKII (Fig. 6F-H).

The results above suggest that Low Extracellular Vesicles (LEVs) could modulate the suppression of proliferation and glycolysis in colon epithelial cells through the SIRT5/p53 pathway, leading to the suppression of colon tumor formation.

### Evaluating the clinical significance of SIRT5 and p53 succinylation in colon cancer: implications for patient prognosis

**Fig. 7:**
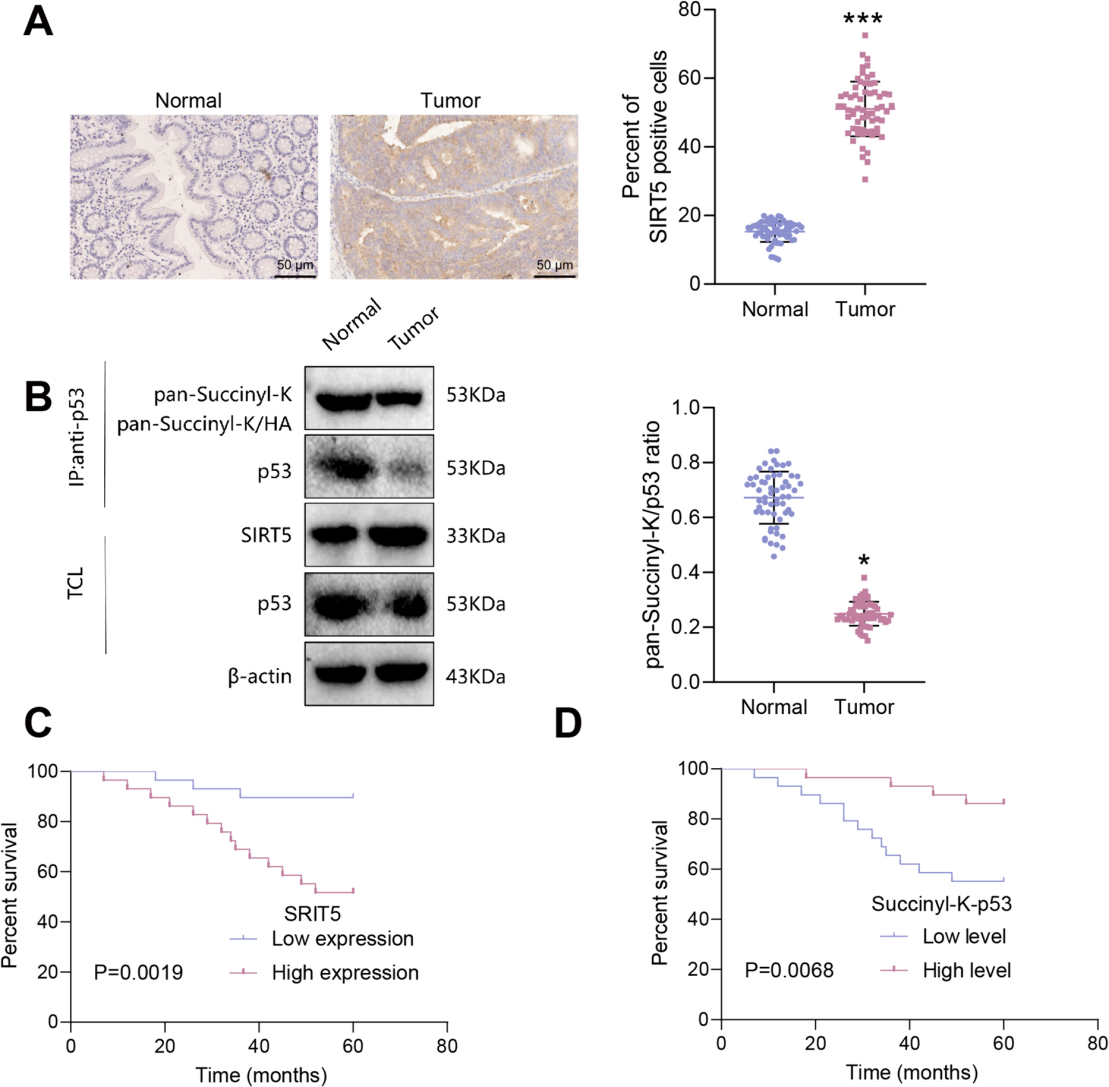
SIRT5 protein and acetylated p53 protein levels in colorectal cancer tissues and their correlation with prognosis. Note: (**A**) Immunohistochemical detection of SIRT5 protein expression in colorectal cancer tissues and adjacent normal tissues (scale bar = 50 μm); (**B**) co-IP experiment detecting the level of acetylated p53 protein in colorectal cancer tissues and adjacent normal tissues; (**C**) Survival curve analysis of SIRT5 protein expression in colorectal cancer tissues and patient prognosis; (**D**) Survival curve analysis of the level of acetylated p53 protein in colorectal cancer tissues and patient prognosis. Normal group, n = 58; Tumor group, n = 58. * represents a difference compared to the Normal group (P < 0.05), *** represents a difference compared to the Normal group (P < 0.001)

Finally, we collected colon cancer tumor tissue and adjacent normal tissue from clinical patients to measure the expression of SIRT5 and p53. We then analyzed the correlation between their expression and patient prognosis. SIRT5 protein expression was detected via immunohistochemistry. The results reveal an increase in SIRT5 protein expression within the Tumor group compared to the Normal group (Fig. 7A). Co-immunoprecipitation (Co-IP) experiments were conducted to assess the level of p53 succinylation. The results demonstrated a decrease in the succinylation level of p53 in the Tumor group compared to the Normal group (Fig. 7B). The survival analysis results indicated that patients exhibiting high expression of SIRT5 protein had a poor prognosis, whereas those with elevated levels of p53 acetylation had a more favorable prognosis (Fig. 7C-D). Subsequently, we explored the impact of SIRT5 on prognosis in TCGA pan-cancer through the Xiantao Academic Bioinformatics website (https://www.xiantaozi.com/). Initially, significant differences in SIRT5 expression levels between 10 cancer tissues—COAD, GBM, HNSC, KIRC, KIRP, LUSC, READ, STAD, THCA, and UCEC—and normal tissues were observed among 33 common cancers (Figure S5A). Assessment of the prognostic impact of SIRT5 in these 10 cancers revealed a correlation between SIRT5 expression levels and prognosis in COAD, KIRC, KIRP, READ, and UCEC (Figure S5B), indicating the significant role of SIRT5 in cancers beyond COAD.

The results above indicate that the SIRT5 protein is highly expressed in cancer tissues of colon cancer patients, whereas the expression level of the p53 succinylation protein is low. This observation is also correlated with patient prognosis.

## Discussion

In recent years, there has been an increase in attention and research focused on the role of probiotics in promoting intestinal health (Wieërs et al. 2020). *Lactobacillus* is a crucial constituent capable of producing advantageous substances like short-chain fatty acids, exerting a positive regulatory impact on the intestinal environment (Zhou et al. 2017). The present study uncovers the potential inhibitory effect of extracellular vesicles (EVs) derived from *Lactobacillus* on the formation of colon cancer, offering novel insights into the specific impact of probiotics on colon cancer.

Previous studies have demonstrated the crucial involvement of the SIRT family and p53 in multiple types of cancer, notably in the regulation of cell cycle and Apoptosis (Wu et al. 2022; Chen et al. 2017; Ong and Ramasamy 2018; Yin et al. 2023). Nevertheless, the role of SIRT5-mediated succinylation of p53 in colon cancer remains unknown (Liu et al. 2022). Our findings offer a novel perspective on comprehending the molecular mechanisms involved in colorectal cancer. It highlights potential therapeutic strategies that focus on targeting this pathway.

Protein acetylation is a distinctive method of protein modification that has recently gained attention (Gao et al. 2021). Previous studies have emphasized the significance of this factor in cardiovascular disease, aging, and metabolic disorders. However, its contribution to cancer remains unclear (Savarese et al. 2023). This study is the first to uncover the role of succinylation in colorectal cancer and offers guidance for future investigations into the association between succinylation and cancer.

The aberrant activation of glycolysis, referred to as the Warburg effect, in cancer has been widely acknowledged. This metabolic reprogramming confers a survival advantage to tumors (Chen and Cubillos-Ruiz 2021). Compared to previous studies, our findings have revealed that external interventions, such as LEVs, can influence the metabolic pathways of cancer cells and offer potential treatment strategies (Hornburg et al. 2021).

The relationship between colitis and colon cancer is well-known (Sato et al. 2023), but distinct from individual studies on SIRT5 (Ekremoglu and Koc 2021; Wang et al. 2020) or p53 research (Liebl and Hofmann 2021), this study confirmed the central role of the SIRT5/p53 axis in this process using a mouse model with p53 gene knockout. This research provides a valuable model for understanding how chronic inflammation promotes colon cancer and offers implications for future clinical practice.

Importantly, this study demonstrates that the expression of SIRT5 and p53 succinylation protein is associated with the prognosis of colon cancer patients, as analyzed using samples obtained from these patients. This study identifies potential biomarkers that could be used in clinical practice and provides evidence supporting therapeutic strategies to target the SIRT5/p53 axis.

**Fig. 8:**
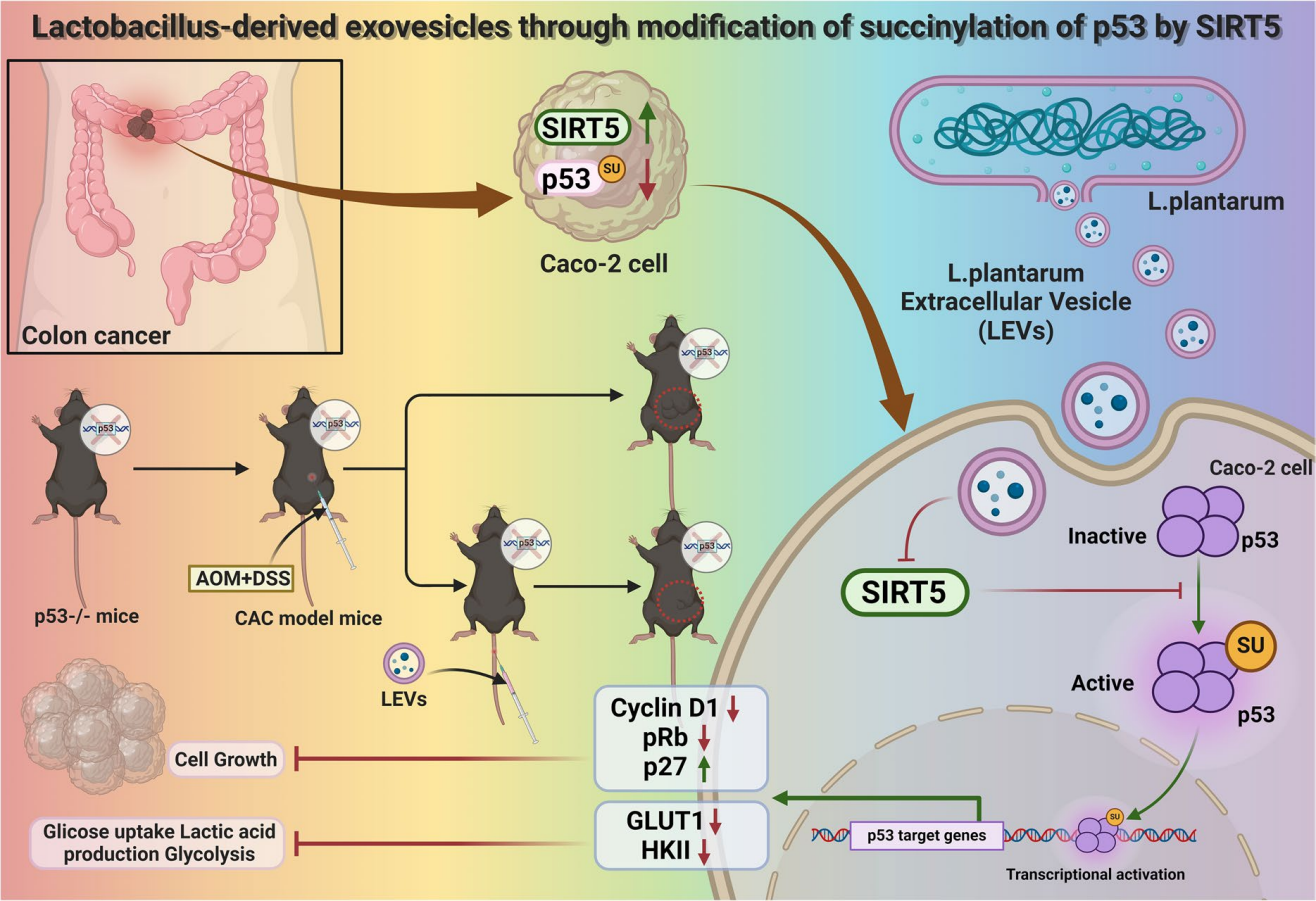
Molecular mechanism diagram illustrating how exosome-like vesicles derived from *Lactobacillus* regulate glycolysis metabolic reprogramming and abnormal proliferation of intestinal epithelial cells through SIRT5-mediated acetylation modification of p53, affecting colon tumor formation

Based on the results above, the following conclusions could be tentatively drawn: Extracellular vesicles derived from *Lactobacillus* inhibit abnormal proliferation and glycolytic reprogramming of intestinal epithelial cells by downregulating SIRT5 and promoting p53 succinylation modification, thereby suppressing the formation of colon tumors (Fig. 8). This study validated SIRT5-mediated desuccinylation of p53 at two levels— *in vivo* animal models and *in vitro* cell experiments—providing deeper insights into the role of protein succinylation modification in colon tumorigenesis, colon epithelial cell proliferation, and glycolytic metabolism. The findings revealed that *Lactobacillus*-derived extracellular vesicles (LEVs) exert an inhibitory effect on colon epithelial cell proliferation and glycolytic metabolism by modulating the SIRT5/p53 axis both *in vivo* and *in vitro*, with no suppressive effect on normal colon epithelial cells (Figure S6). Additionally, the study highlights the significance of *Lactobacillus* (Heeney et al. 2018; Wang et al. 2022) in intestinal health and disease prevention, suggesting the therapeutic potential of *Lactobacillus*-based treatments against colon cancer.

Furthermore, we validated the correlation between succinylation levels of SIRT5 and p53 and the prognosis of colon cancer patients using clinical tissue samples. Additionally, the correlation between SIRT5 and the prognosis of colon cancer patients was further confirmed through bioinformatics analysis. The study also confirmed the prognostic relevance of SIRT5 in other types of cancer, suggesting its potential applicability across various cancer types. However, there are limitations to this study. Due to constraints, we did not further investigate the mechanistic role of SIRT5 and p53 in other cancers experimentally. Moreover, while the research revealed that *Lactobacillus*-derived extracellular vesicles inhibit colon epithelial cell proliferation and glycolytic metabolism through the SIRT5/p53 axis, the specific underlying mechanism of this process requires further exploration. For instance, understanding how Lactobacillus-derived extracellular vesicles influence the interaction between SIRT5 and p53, and how this influence leads to the reprogramming of glycolytic metabolism and suppression of cell proliferation, are crucial questions for our future research endeavors.

## Supplementary Information

Below is the link to the electronic supplementary material.

Supplementary file1 Figure S1. Growth and pH changes of L.plantarum at different times in MRS broth. Note: (A) The curve showing the changes in bacterial count overtime during the growth of L.plantarum in MRS; (B) The curve showing the changes in pH value over time during the growth of L.plantarum. (JPG 565 KB)

Supplementary file2 Figure S2. LEVs regulate SIRT5 expression, affecting proliferation and glycolysis metabolism of colorectal cancer cells SW480. Note: (A-B) RT-qPCR and Western blot detecting the mRNA and protein expression of SIRT5 in SW480 cells after SIRT5 overexpression; (C-D) RT-qPCR and Western blot detecting the mRNA and protein expression of SIRT5 in different groups of SW480 cells; (E) EdU staining detecting the proliferation of SW480 cells in each group (scale bar = 25 μm); (F) Colony formation assay detecting the colony formation ability of SW480 cells in each group; (G) Flow cytometry detecting cell cycle changes in SW480 cells in each group; (H) Western blot detecting the expression changes of cell cycle-related proteins in SW480 cells in each group; (I) Glucose uptake in SW480 cells in each group; (J) Lactate production in SW480 cells in each group; (K) Western blot detecting the expression of glycolytic rate-limiting enzymes in SW480 cells in each group. * represents a difference compared to the oe-NC or PBS group (P < 0.05), # represents a difference compared to the LEVs+oe-NC group (P < 0.05), experiments repeated 3 times. (JPG 1779 KB)

Supplementary file3 Figure S3. Interactions between SIRT5 and p53. Note: (A) KEGG pathway enrichment analysis of differentially expressed genes obtained from Caco-2 cells treated with LEVs; (B) Protein interaction network of candidate target genes encoded by p53 (TP53) and their encoded proteins. (JPG 1588 KB)

Supplementary file4 Figure S4. LEVs regulate the SIRT5/p53 axis, affecting the proliferation and glycolysis metabolism of colorectal cancer cells SW480. Note: (A) co-IP experiment detecting the level of acetylated p53 protein in different groups of SW480 cells; (B) EdU staining detecting the proliferation of SW480 cells in each group (scale bar = 25 μm); (C) Colony formation assay detecting the colony formation ability of SW480 cells in each group; (D) Flow cytometry detecting cell cycle changes in SW480 cells in each group; (E) Western blot detecting the expression changes of cell cycle-related proteins in SW480 cells in each group; (F) Glucose uptake in SW480 cells in each group; (G) Lactate production in SW480 cells in each group; (H-I) Western blot detecting the expression of glycolytic rate-limiting enzymes in SW480 cells in each group. * represents a difference compared to the Control group (P < 0.05), # represents a difference compared to the LEVs+sh-NC group (P < 0.05), experiments repeated 3 times. (JPG 1177 KB)

Supplementary file5 Figure S5. Expression Levels of SIRT5 in Other Cancers and Its Correlation with Prognosis. Note: (A) SIRT5 expression levels in 33 cancer tissues and normal tissues; (B) Protein expression of SIRT5 and survival curve analysis of patients with COAD, GBM, HNSC, KIRC, KIRP, LUSC, READ, STAD, THCA, and UCEC; * indicates P < 0.05 compared to the Normal group; ** indicates P < 0.01 compared to the Normal group; *** indicates P < 0.001 compared to the Normal group. (JPG 1330 KB)

Supplementary file6 Figure S6 LEVs inhibit NCM460 cell growth. NCM460 cells were treated with isolated LEVs, followed by (A) EdU staining to assess proliferation of various groups of Caco-2 cells (scale bar=25 μm); (B) colony formation assay to evaluate the clonogenic ability of different groups of Caco-2 cells, with the cell experiment repeated three times. (JPG 1145 KB)

Supplementary file7 (DOCX 12 KB)

## Data Availability

The data that supports the findings of this study are available on request from the corresponding author upon reasonable request.

## References

[CR1] Aiello P, Sharghi M, Mansourkhani SM, et al. Medicinal Plants in the Prevention and Treatment of Colon Cancer. Oxid Med Cell Longev. 2075614. 10.1155/2019/2075614. 10.1155/2019/2075614 PMC7187726 32377288

[CR2] Antropova EA, Khlebodarova TM, Demenkov PS, et al. Computer analysis of regulation of hepatocarcinoma marker genes hypermethylated by HCV proteins. Vavilovskii Zhurnal Genet Selektsii. 2022;26(8):733–42. 10.18699/VJGB-22-89. 36714033 10.18699/VJGB-22-89 PMC9840909

[CR3] Bajic SS, Cañas MA, Tolinacki M, et al. Proteomic profile of extracellular vesicles released by Lactiplantibacillus plantarum BGAN8 and their internalization by non-polarized HT29 cell line. Sci Rep. 2020;10(1):21829. 10.1038/s41598-020-78920-z. 33311536 10.1038/s41598-020-78920-z PMC7732981

[CR4] Bao Y, Zhai J, Chen H, et al. Targeting m^6^A reader YTHDF1 augments antitumour immunity and boosts anti-PD-1 efficacy in colorectal cancer. Gut. 2023;72(8):1497–509. 10.1136/gutjnl-2022-328845. 36717220 10.1136/gutjnl-2022-328845 PMC10359538

[CR5] Bauer DE, Harris MH, Plas DR, et al. Cytokine stimulation of aerobic glycolysis in hematopoietic cells exceeds proliferative demand. FASEB J. 2004;18(11):1303–5. 10.1096/fj.03-1001fje. 15180958 10.1096/fj.03-1001fje PMC4458073

[CR6] Briaud P, Carroll RK. Extracellular Vesicle Biogenesis and Functions in Gram-Positive Bacteria. Infect Immun. 2020;88(12):e00433-20. 10.1128/IAI.00433-20. 32989035 10.1128/IAI.00433-20 PMC7671900

[CR7] Bu D, Luo H, Huo P, et al. KOBAS-i: intelligent prioritization and exploratory visualization of biological functions for gene enrichment analysis. Nucleic Acids Res. 2021;49(W1):W317–25. 10.1093/nar/gkab447. 34086934 10.1093/nar/gkab447 PMC8265193

[CR8] Chee WJY, Chew SY, Than LTL. Vaginal microbiota and the potential of Lactobacillus derivatives in maintaining vaginal health. Microb Cell Fact. 2020;19(1):203. 10.1186/s12934-020-01464-4. 33160356 10.1186/s12934-020-01464-4 PMC7648308

[CR9] Chen X, Cubillos-Ruiz JR. Endoplasmic reticulum stress signals in the tumour and its microenvironment. Nat Rev Cancer. 2021;21(2):71–88. 10.1038/s41568-020-00312-2. 33214692 10.1038/s41568-020-00312-2 PMC7927882

[CR10] Chen S, Shen X. Long noncoding RNAs: functions and mechanisms in colon cancer. Mol Cancer. 2020;19(1):167. 10.1186/s12943-020-01287-2. 33246471 10.1186/s12943-020-01287-2 PMC7697375

[CR11] Chen J, Wang A, Chen Q. SirT3 and p53 Deacetylation in Aging and Cancer. J Cell Physiol. 2017;232(9):2308–11. 10.1002/jcp.25669. 27791271 10.1002/jcp.25669

[CR12] Chen D, Bao C, Zhao F, et al. Exploring Specific miRNA-mRNA Axes With Relationship to Taxanes-Resistance in Breast Cancer. Front Oncol. 2020;10:1397. 10.3389/fonc.2020.01397. 32974144 10.3389/fonc.2020.01397 PMC7473300

[CR13] Chen Y, Jiang Y, Huang X, et al. Cordycepin Inhibits the Growth of Hepatocellular Carcinoma by Regulating the Pathway of Aerobic Glycolysis. Evid Based Complement Alternat Med. 2022;2022:6454482. 10.1155/2022/6454482. 36467556 10.1155/2022/6454482 PMC9711956

[CR14] Cheng X, Zhang G, Zhang L, et al. Mesenchymal stem cells deliver exogenous miR-21 via exosomes to inhibit nucleus pulposus cell apoptosis and reduce intervertebral disc degeneration. J Cell Mol Med. 2018;22(1):261–76. 10.1111/jcmm.13316. 28805297 10.1111/jcmm.13316 PMC5742691

[CR15] Corrigendum. Genome. Biol Evol. 2020;12(4):493. 10.1093/gbe/evaa066. 10.1093/gbe/evaa066 PMC7192530 32353151

[CR16] Dai X, Lu L, Deng S, et al. USP7 targeting modulates anti-tumor immune response by reprogramming Tumor-associated Macrophages in Lung Cancer. Theranostics. 2020;10(20):9332–47. 10.7150/thno.47137. 32802195 10.7150/thno.47137 PMC7415808

[CR17] Das UN. Cell Membrane Theory of Senescence" and the Role of Bioactive Lipids in Aging, and Aging Associated Diseases and Their Therapeutic Implications. Biomolecules. 2021;11(2):241. 10.3390/biom11020241. 33567774 10.3390/biom11020241 PMC7914625

[CR18] DeBerardinis RJ, Lum JJ, Hatzivassiliou G, Thompson CB. The biology of cancer: metabolic reprogramming fuels cell growth and proliferation. Cell Metab. 2008;7(1):11–20. 10.1016/j.cmet.2007.10.002. 18177721 10.1016/j.cmet.2007.10.002

[CR19] Ekremoglu O, Koc A. The role of SIRT5 and p53 proteins in the sensitivity of colon cancer cells to chemotherapeutic agent 5-Fluorouracil. Mol Biol Rep. 2021;48(7):5485–95. 10.1007/s11033-021-06558-9. 34279763 10.1007/s11033-021-06558-9

[CR20] Fabregas JC, Ramnaraign B, George TJ. Clinical Updates for Colon Cancer Care in 2022. Clin Colorectal Cancer. 2022;21(3):198–203. 10.1016/j.clcc.2022.05.006. 35729033 10.1016/j.clcc.2022.05.006

[CR21] Fan Q, Yang L, Zhang X, et al. Autophagy promotes metastasis and glycolysis by upregulating MCT1 expression and Wnt/β-catenin signaling pathway activation in hepatocellular carcinoma cells. J Exp Clin Cancer Res. 2018;37(1):9. 10.1186/s13046-018-0673-y. 29351758 10.1186/s13046-018-0673-y PMC5775607

[CR22] Gao Y, Zhu Y, Tran EL, et al. MnSOD Lysine 68 acetylation leads to cisplatin and doxorubicin resistance due to aberrant mitochondrial metabolism. Int J Biol Sci. 2021;17(5):1203–16. 10.7150/ijbs.51184. 33867840 10.7150/ijbs.51184 PMC8040469

[CR23] Hao H, Zhang X, Tong L, et al. Effect of Extracellular Vesicles Derived From Lactobacillus plantarum Q7 on Gut Microbiota and Ulcerative Colitis in Mice. Front Immunol. 2021;12:777147. 10.3389/fimmu.2021.777147. 34925349 10.3389/fimmu.2021.777147 PMC8674835

[CR24] Heeney DD, Gareau MG, Marco ML. Intestinal Lactobacillus in health and disease, a driver or just along for the ride? Curr Opin Biotechnol. 2018;49:140–7. 10.1016/j.copbio.2017.08.004. 28866243 10.1016/j.copbio.2017.08.004 PMC5808898

[CR25] Hornburg M, Desbois M, Lu S, et al. Single-cell dissection of cellular components and interactions shaping the tumor immune phenotypes in ovarian cancer. Cancer Cell. 2021;39(7):928-944.e6. 10.1016/j.ccell.2021.04.004. 33961783 10.1016/j.ccell.2021.04.004

[CR26] Islam MR, Akash S, Rahman MM, et al. Colon cancer and colorectal cancer: Prevention and treatment by potential natural products. Chem Biol Interact. 2022;368: 110170. 10.1016/j.cbi.2022.110170. 36202214 10.1016/j.cbi.2022.110170

[CR27] Jackson DN, Theiss AL. Gut bacteria signaling to mitochondria in intestinal inflammation and cancer. Gut Microbes. 2020;11(3):285–304. 10.1080/19490976.2019.1592421. 30913966 10.1080/19490976.2019.1592421 PMC7524274

[CR28] Jin W, Liao X, Lv Y, et al. MUC1 induces acquired chemoresistance by upregulating ABCB1 in EGFR-dependent manner. Cell Death Dis. 2017;8(8):e2980. 10.1038/cddis.2017.378. 28796259 10.1038/cddis.2017.378 PMC5596566

[CR29] Jin D, Guo J, Wu Y, et al. m^6^A mRNA methylation initiated by METTL3 directly promotes YAP translation and increases YAP activity by regulating the MALAT1-miR-1914–3p-YAP axis to induce NSCLC drug resistance and metastasis [retracted in: J Hematol Oncol. 2023 Feb 22;16(1):14]. J Hematol Oncol. 2019;12(1):135. Published 2019 Dec 9. 10.1186/s13045-019-0830-6. 10.1186/s13045-023-01416-6 PMC9948469 36814345

[CR30] Kim W, Lee EJ, Bae IH, et al. Lactobacillus plantarum-derived extracellular vesicles induce anti-inflammatory M2 macrophage polarization in vitro. J Extracell Vesicles. 2020;9(1):1793514. 10.1080/20013078.2020.1793514. 32944181 10.1080/20013078.2020.1793514 PMC7480564

[CR31] Kim K, Park J, Sohn Y, et al. Stability of Plant Leaf-Derived Extracellular Vesicles According to Preservative and Storage Temperature. Pharmaceutics. 2022;14(2):457. 10.3390/pharmaceutics14020457. 35214189 10.3390/pharmaceutics14020457 PMC8879201

[CR32] Kwon OK, Bang IH, Choi SY, et al. LDHA Desuccinylase Sirtuin 5 as A Novel Cancer Metastatic Stimulator in Aggressive Prostate Cancer. Genomics Proteomics Bioinformatics. 2023;21(1):177–89. 10.1016/j.gpb.2022.02.004. 35278714 10.1016/j.gpb.2022.02.004 PMC10372916

[CR33] Lahmidani N, Miry S, Abid H, et al. Gastric Adenocarcinoma in a Moroccan Population: First Report on Survival Data. Gulf J Oncolog. 2019;1(31):36–40. 31591989

[CR34] Lee BH, Wu SC, Shen TL, Hsu YY, Chen CH, Hsu WH. The applications of Lactobacillus plantarum-derived extracellular vesicles as a novel natural antibacterial agent for improving quality and safety in tuna fish. Food Chem. 2021;340: 128104. 10.1016/j.foodchem.2020.128104. 33010644 10.1016/j.foodchem.2020.128104

[CR35] Li L, Li Y, Lin J, et al. Phosphorylated Myosin Light Chain 2 (p-MLC2) as a Molecular Marker of Antemortem Coronary Artery Spasm. Med Sci Monit. 2016;22:3316–27. 10.12659/msm.900152. 27643564 10.12659/MSM.900152 PMC5031170

[CR36] Li M, Lee K, Hsu M, Nau G, Mylonakis E, Ramratnam B. Lactobacillus-derived extracellular vesicles enhance host immune responses against vancomycin-resistant enterococci. BMC Microbiol. 2017;17(1):66. 10.1186/s12866-017-0977-7. 28288575 10.1186/s12866-017-0977-7 PMC5348868

[CR37] Liebl MC, Hofmann TG. The Role of p53 Signaling in Colorectal Cancer. Cancers (Basel). 2021;13(9):2125. 10.3390/cancers13092125. 33924934 10.3390/cancers13092125 PMC8125348

[CR38] Lightfoot YL, Yang T, Sahay B, Mohamadzadeh M. Targeting aberrant colon cancer-specific DNA methylation with lipoteichoic acid-deficient Lactobacillus acidophilus. Gut Microbes. 2013;4(1):84–8. 10.4161/gmic.22822. 23137966 10.4161/gmic.22822 PMC3555892

[CR39] Lin JE, Li P, Snook AE, et al. The hormone receptor GUCY2C suppresses intestinal tumor formation by inhibiting AKT signaling. Gastroenterology. 2010;138(1):241–54. 10.1053/j.gastro.2009.08.064. 19737566 10.1053/j.gastro.2009.08.064 PMC2813361

[CR40] Liu Y, Gu W. The complexity of p53-mediated metabolic regulation in tumor suppression. Semin Cancer Biol. 2022;85:4–32. 10.1016/j.semcancer.2021.03.010. 33785447 10.1016/j.semcancer.2021.03.010 PMC8473587

[CR41] Liu J, Lichtenberg T, Hoadley KA, et al. An Integrated TCGA Pan-Cancer Clinical Data Resource to Drive High-Quality Survival Outcome Analytics. Cell. 2018;173(2):400-416.e11. 10.1016/j.cell.2018.02.052. 29625055 10.1016/j.cell.2018.02.052 PMC6066282

[CR42] Liu X, Rong F, Tang J, et al. Repression of p53 function by SIRT5-mediated desuccinylation at Lysine 120 in response to DNA damage. Cell Death Differ. 2022;29(4):722–36. 10.1038/s41418-021-00886-w. 34642466 10.1038/s41418-021-00886-w PMC8989948

[CR43] Liu X, Qin H, Zhang L, et al. Hyperoxia induces glucose metabolism reprogramming and intracellular acidification by suppressing MYC/MCT1 axis in lung cancer. Redox Biol. 2023;61: 102647. 10.1016/j.redox.2023.102647. 36867943 10.1016/j.redox.2023.102647 PMC10011425

[CR44] Lu L, Mullins CS, Schafmayer C, Zeißig S, Linnebacher M. A global assessment of recent trends in gastrointestinal cancer and lifestyle-associated risk factors. Cancer Commun (lond). 2021;41(11):1137–51. 10.1002/cac2.12220. 34563100 10.1002/cac2.12220 PMC8626600

[CR45] Ma F, Sun M, Song Y, et al. Lactiplantibacillus plantarum-12 Alleviates Inflammation and Colon Cancer Symptoms in AOM/DSS-Treated Mice through Modulating the Intestinal Microbiome and Metabolome. Nutrients. 2022;14(9):1916. 10.3390/nu14091916. 35565884 10.3390/nu14091916 PMC9100115

[CR46] Mao L, Xin F, Ren J, et al. 5-HT2B-mediated serotonin activation in enterocytes suppresses colitis-associated cancer initiation and promotes cancer progression. Theranostics. 2022;12(8):3928–45. 10.7150/thno.70762. 35664068 10.7150/thno.70762 PMC9131283

[CR47] Meng Y, Xu X, Luan H, et al. The progress and development of GLUT1 inhibitors targeting cancer energy metabolism. Future Med Chem. 2019;11(17):2333–52. 10.4155/fmc-2019-0052. 31581916 10.4155/fmc-2019-0052

[CR48] Miura T, Ohtsuka H, Aoki T, et al. Increased neutrophil-lymphocyte ratio predicts recurrence in patients with well-differentiated pancreatic neuroendocrine neoplasm based on the 2017 World Health Organization classification. BMC Surg. 2021;21(1):176. 10.1186/s12893-021-01178-3. 33789657 10.1186/s12893-021-01178-3 PMC8011407

[CR49] Moreno-Sánchez R, Rodríguez-Enríquez S, Marín-Hernández A, Saavedra E. Energy metabolism in tumor cells. FEBS J. 2007;274(6):1393–418. 10.1111/j.1742-4658.2007.05686.x. 17302740 10.1111/j.1742-4658.2007.05686.x

[CR50] Naureen Z, Medori MC, Dhuli K, et al. Polyphenols and Lactobacillus reuteri in oral health. J Prev Med Hyg. 2022;63(2 Suppl 3):E246–54. 10.15167/2421-4248/jpmh2022.63.2S3.2767. 36479495 10.15167/2421-4248/jpmh2022.63.2S3.2767 PMC9710395

[CR51] Neufert C, Becker C, Neurath MF. An inducible mouse model of colon carcinogenesis for the analysis of sporadic and inflammation-driven tumor progression. Nat Protoc. 2007;2(8):1998–2004. 10.1038/nprot.2007.279. 17703211 10.1038/nprot.2007.279

[CR52] Ong ALC, Ramasamy TS. Role of Sirtuin1-p53 regulatory axis in aging, cancer and cellular reprogramming. Ageing Res Rev. 2018;43:64–80. 10.1016/j.arr.2018.02.004. 29476819 10.1016/j.arr.2018.02.004

[CR53] Otani K, Kawai K, Hata K, et al. Colon cancer with perforation. Surg Today. 2019;49(1):15–20. 10.1007/s00595-018-1661-8. 29691659 10.1007/s00595-018-1661-8

[CR54] Qin Y, Havulinna AS, Liu Y, et al. Combined effects of host genetics and diet on human gut microbiota and incident disease in a single population cohort. Nat Genet. 2022;54(2):134–42. 10.1038/s41588-021-00991-z. 35115689 10.1038/s41588-021-00991-z PMC9883041

[CR55] Regan JL, Schumacher D, Staudte S, et al. RNA sequencing of long-term label-retaining colon cancer stem cells identifies novel regulators of quiescence. iScience. 2021;24(6):102618. 10.1016/j.isci.2021.102618. 34142064 10.1016/j.isci.2021.102618 PMC8185225

[CR56] Roslan NH, Makpol S, Mohd Yusof YA. A Review on Dietary Intervention in Obesity Associated Colon Cancer. Asian Pac J Cancer Prev. 2019;20(5):1309–19. 10.31557/APJCP.2019.20.5.1309. 31127882 10.31557/APJCP.2019.20.5.1309 PMC6857900

[CR57] Salem M, Shan Y, Bernaudo S, Peng C. miR-590–3p Targets Cyclin G2 and FOXO3 to Promote Ovarian Cancer Cell Proliferation, Invasion, and Spheroid Formation. Int J Mol Sci. 2019;20(8):1810. 10.3390/ijms20081810. 31013711 10.3390/ijms20081810 PMC6515004

[CR58] Sato Y, Tsujinaka S, Miura T, Kitamura Y, Suzuki H, Shibata C. Inflammatory Bowel Disease and Colorectal Cancer: Epidemiology, Etiology, Surveillance, and Management. Cancers (Basel). 2023;15(16):4154. 10.3390/cancers15164154. 37627182 10.3390/cancers15164154 PMC10452690

[CR59] Savarese G, Becher PM, Lund LH, Seferovic P, Rosano GMC, Coats AJS. Global burden of heart failure: a comprehensive and updated review of epidemiology. Cardiovasc Res. 2023;118(17):3272–87. 10.1093/cvr/cvac013. 35150240 10.1093/cvr/cvac013

[CR60] Saxami G, Karapetsas A, Chondrou P, et al. Potentially probiotic Lactobacillus strains with anti-proliferative activity induce cytokine/chemokine production and neutrophil recruitment in mice. Benef Microbes. 2017;8(4):615–23. 10.3920/BM2016.0202. 28618861 10.3920/BM2016.0202

[CR61] Shen C, Li J, Chang S, Che G. Zhongguo Fei Ai Za Zhi. 2020;23(10):921–6. 10.3779/j.issn.1009-3419.2020.101.32. 33070516 10.3779/j.issn.1009-3419.2020.101.32 PMC7583875

[CR62] Shu M, Zheng X, Wu S, et al. Targeting oncogenic miR-335 inhibits growth and invasion of malignant astrocytoma cells. Mol Cancer. 2011;10:59. 10.1186/1476-4598-10-59. 21592405 10.1186/1476-4598-10-59 PMC3129318

[CR63] Song M, Chan AT, Sun J. Influence of the Gut Microbiome, Diet, and Environment on Risk of Colorectal Cancer. Gastroenterology. 2020;158(2):322–40. 10.1053/j.gastro.2019.06.048. 31586566 10.1053/j.gastro.2019.06.048 PMC6957737

[CR64] Sung JY, Cheong JH. Pan-Cancer Analysis Reveals Distinct Metabolic Reprogramming in Different Epithelial-Mesenchymal Transition Activity States. Cancers (Basel). 2021;13(8):1778. 10.3390/cancers13081778. 33917859 10.3390/cancers13081778 PMC8068218

[CR65] Sung JY, Cheong JH. Intercellular communications and metabolic reprogramming as new predictive markers for immunotherapy responses in gastric cancer. Cancer Commun (lond). 2022a;42(6):572–5. 10.1002/cac2.12285. 35396921 10.1002/cac2.12285 PMC9198349

[CR66] Sung JY, Cheong JH. New Immunometabolic Strategy Based on Cell Type-Specific Metabolic Reprogramming in the Tumor Immune Microenvironment. Cells. 2022;11(5):768. 10.3390/cells11050768. 35269390 10.3390/cells11050768 PMC8909366

[CR67] Tian J, Yuan L. Sirtuin 6 inhibits colon cancer progression by modulating PTEN/AKT signaling. Biomed Pharmacother. 2018;106:109–16. 10.1016/j.biopha.2018.06.070. 29957460 10.1016/j.biopha.2018.06.070

[CR68] Velazquez R, Ferreira E, Knowles S, et al. Lifelong choline supplementation ameliorates Alzheimer’s disease pathology and associated cognitive deficits by attenuating microglia activation. Aging Cell. 2019;18(6): e13037. 10.1111/acel.13037. 31560162 10.1111/acel.13037 PMC6826123

[CR69] Wang K, Hu Z, Zhang C, et al. SIRT5 Contributes to Colorectal Cancer Growth by Regulating T Cell Activity. J Immunol Res. 2020;2020:3792409. 10.1155/2020/3792409. 32953892 10.1155/2020/3792409 PMC7481950

[CR70] Wang X, Huang J, Chen W, Li G, Li Z, Lei J. The updated role of exosomal proteins in the diagnosis, prognosis, and treatment of cancer. Exp Mol Med. 2022;54(9):1390–400. 10.1038/s12276-022-00855-4. 36138197 10.1038/s12276-022-00855-4 PMC9535014

[CR71] Wang W, Lu K, Jiang X, et al. Ferroptosis inducers enhanced cuproptosis induced by copper ionophores in primary liver cancer. J Exp Clin Cancer Res. 2023;42(1):142. 10.1186/s13046-023-02720-2. 37277863 10.1186/s13046-023-02720-2 PMC10242978

[CR72] Wei S, Peng L, Yang J, et al. Exosomal transfer of miR-15b-3p enhances tumorigenesis and malignant transformation through the DYNLT1/Caspase-3/Caspase-9 signaling pathway in gastric cancer. J Exp Clin Cancer Res. 2020;39(1):32. 10.1186/s13046-019-1511-6. 32039741 10.1186/s13046-019-1511-6 PMC7011526

[CR73] Wieërs G, Belkhir L, Enaud R, et al. How Probiotics Affect the Microbiota. Front Cell Infect Microbiol. 2020;9:454. 10.3389/fcimb.2019.00454. 32010640 10.3389/fcimb.2019.00454 PMC6974441

[CR74] Wu QJ, Zhang TN, Chen HH, et al. The sirtuin family in health and disease. Signal Transduct Target Ther. 2022;7(1):402. 10.1038/s41392-022-01257-8. 36581622 10.1038/s41392-022-01257-8 PMC9797940

[CR75] Xian P, Hei Y, Wang R, et al. Mesenchymal stem cell-derived exosomes as a nanotherapeutic agent for amelioration of inflammation-induced astrocyte alterations in mice. Theranostics. 2019;9(20):5956–75. 10.7150/thno.33872. 31534531 10.7150/thno.33872 PMC6735367

[CR76] Xiang DM, Sun W, Zhou T, et al. Oncofetal HLF transactivates c-Jun to promote hepatocellular carcinoma development and sorafenib resistance. Gut. 2019;68(10):1858–71. 10.1136/gutjnl-2018-317440. 31118247 10.1136/gutjnl-2018-317440

[CR77] Xu Y. Regulation of p53 responses by post-translational modifications. Cell Death Differ. 2003;10(4):400–3. 10.1038/sj.cdd.4401182. 12719715 10.1038/sj.cdd.4401182

[CR78] Yang X, Wang Z, Li X, et al. SHMT2 Desuccinylation by SIRT5 Drives Cancer Cell Proliferation. Cancer Res. 2018;78(2):372–86. 10.1158/0008-5472.CAN-17-1912. 29180469 10.1158/0008-5472.CAN-17-1912

[CR79] Yang R, Huang H, Cui S, Zhou Y, Zhang T, Zhou Y. IFN-γ promoted exosomes from mesenchymal stem cells to attenuate colitis via miR-125a and miR-125b. Cell Death Dis. 2020;11(7):603. 10.1038/s41419-020-02788-0. 32733020 10.1038/s41419-020-02788-0 PMC7393506

[CR80] Yang Z, Gao Z, Yang Z, et al. Lactobacillus plantarum-derived extracellular vesicles protect against ischemic brain injury via the microRNA-101a-3p/c-Fos/TGF-β axis. Pharmacol Res. 2022;182: 106332. 10.1016/j.phrs.2022.106332. 35779817 10.1016/j.phrs.2022.106332

[CR81] Yao Y, Liu C, Wang B, et al. HOXB9 blocks cell cycle progression to inhibit pancreatic cancer cell proliferation through the DNMT1/RBL2/c-Myc axis. Cancer Lett. 2022;533: 215595. 10.1016/j.canlet.2022.215595. 35182659 10.1016/j.canlet.2022.215595

[CR82] Ye M, Dong S, Hou H, Zhang T, Shen M. Oncogenic Role of Long Noncoding RNAMALAT1 in Thyroid Cancer Progression through Regulation of the miR-204/IGF2BP2/m6A-MYC Signaling. Mol Ther Nucleic Acids. 2020;23:1–12. 10.1016/j.omtn.2020.09.023. 33312756 10.1016/j.omtn.2020.09.023 PMC7711188

[CR83] Yin JY, Lu XT, Hou ML, Cao T, Tian Z. Sirtuin1-p53: A potential axis for cancer therapy. Biochem Pharmacol. 2023;212: 115543. 10.1016/j.bcp.2023.115543. 37037265 10.1016/j.bcp.2023.115543

[CR84] Yue Y, Ye K, Lu J, et al. Probiotic strain Lactobacillus plantarum YYC-3 prevents colon cancer in mice by regulating the tumour microenvironment. Biomed Pharmacother. 2020a;127: 110159. 10.1016/j.biopha.2020.110159. 32353824 10.1016/j.biopha.2020.110159

[CR85] Yue YC, Yang BY, Lu J, et al. Metabolite secretions of Lactobacillus plantarum YYC-3 may inhibit colon cancer cell metastasis by suppressing the VEGF-MMP2/9 signaling pathway. Microb Cell Fact. 2020;19(1):213. 10.1186/s12934-020-01466-2. 33228670 10.1186/s12934-020-01466-2 PMC7684877

[CR86] Zhang H, Gao Q, Tan S, et al. SET8 prevents excessive DNA methylation by methylation-mediated degradation of UHRF1 and DNMT1. Nucleic Acids Res. 2019a;47(17):9053–68. 10.1093/nar/gkz626. 31400111 10.1093/nar/gkz626 PMC6753495

[CR87] Zhang L, Chen J, Ning D, et al. FBXO22 promotes the development of hepatocellular carcinoma by regulating the ubiquitination and degradation of p21. J Exp Clin Cancer Res. 2019;38(1):101. 10.1186/s13046-019-1058-6. 30808376 10.1186/s13046-019-1058-6 PMC6390379

[CR88] Zhou L, Zhao Y. B7–H3 Induces Ovarian Cancer Drugs Resistance Through An PI3K/AKT/BCL-2 Signaling Pathway. Cancer Manag Res. 2019;11:10205–14. 10.2147/CMAR.S222224. 31819652 10.2147/CMAR.S222224 PMC6899073

[CR89] Zhou D, Pan Q, Xin FZ, et al. Sodium butyrate attenuates high-fat diet-induced steatohepatitis in mice by improving gut microbiota and gastrointestinal barrier. World J Gastroenterol. 2017;23(1):60–75. 10.3748/wjg.v23.i1.60. 28104981 10.3748/wjg.v23.i1.60 PMC5221287

