# Supplementary figures and images for: Antitumorigenic potential of Lactobacillus-derived extracellular vesicles: p53 succinylation and glycolytic reprogramming in intestinal epithelial cells via SIRT5 modulation
Source: Cell Biol Toxicol. 2024 Aug 7;40(1):66. doi: 10.1007/s10565-024-09897-y (PMC11306434; doi:10.1007/s10565-024-09897-y)

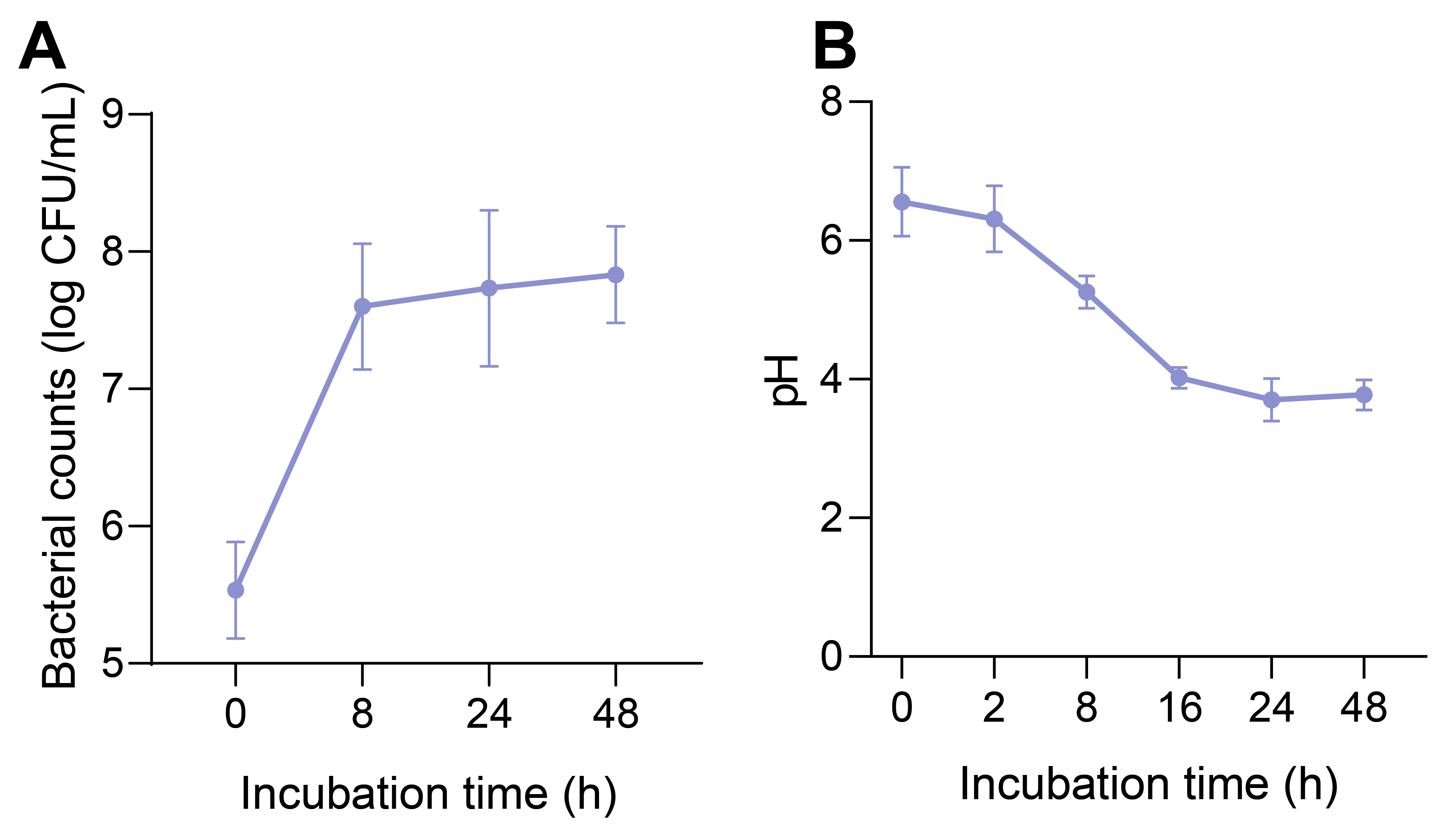

Supplement: Supplementary file 1 — Supplementary file1 Figure S1. Growth and pH changes of L.plantarum at different times in MRS broth. Note: (A) The curve showing the changes in bacterial count overtime during the growth of L.plantarum in MRS; (B) The curve showing the changes in pH value over time during the growth of L.plantarum. (JPG 565 KB) [file 10565_2024_9897_MOESM1_ESM.jpg]

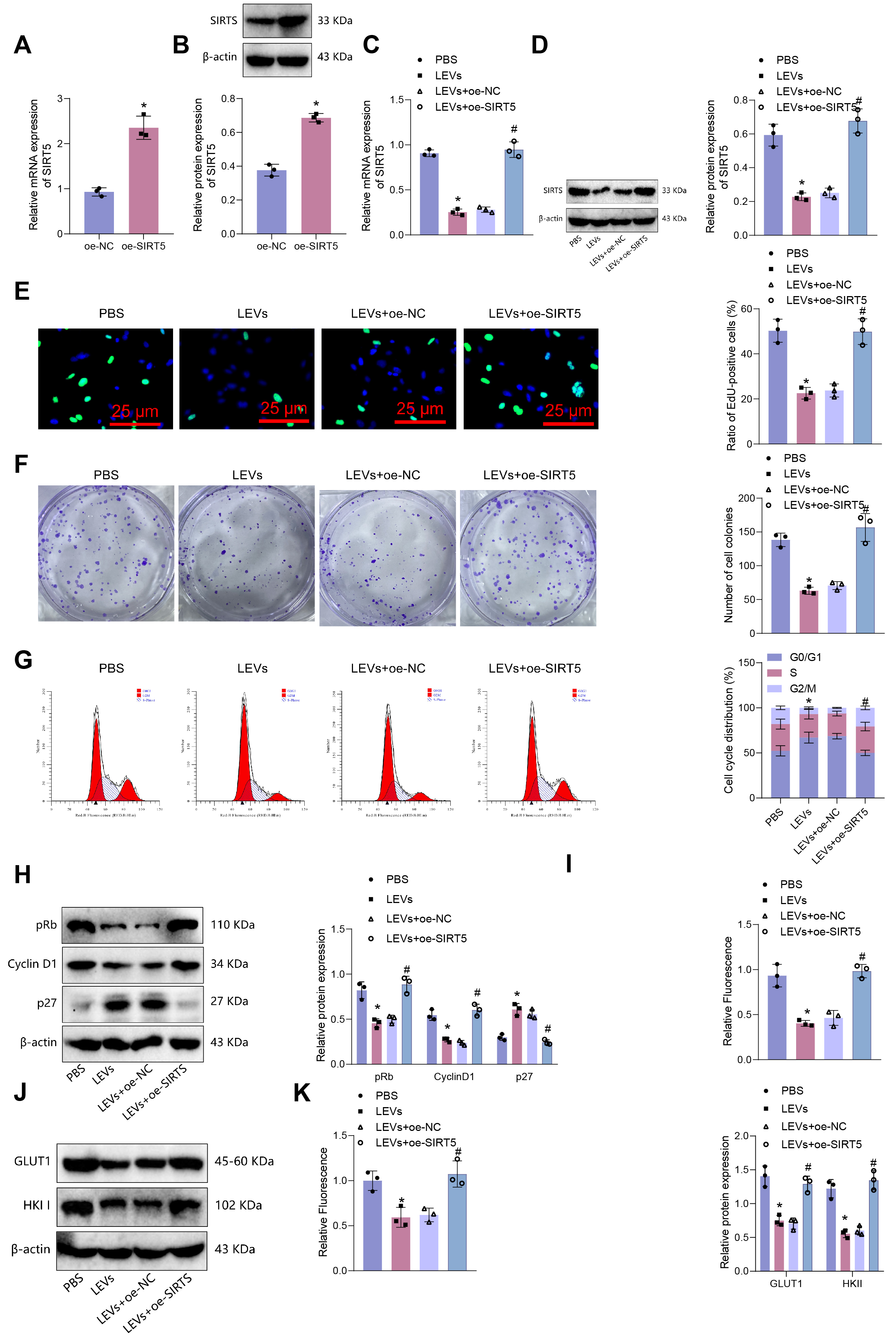

Supplement: Supplementary file 2 — Supplementary file2 Figure S2. LEVs regulate SIRT5 expression, affecting proliferation and glycolysis metabolism of colorectal cancer cells SW480. Note: (A-B) RT-qPCR and Western blot detecting the mRNA and protein expression of SIRT5 in SW480 cells after SIRT5 overexpression; (C-D) RT-qPCR and Western blot detecting the mRNA and protein expression of SIRT5 in different groups of SW480 cells; (E) EdU staining detecting the proliferation of SW480 cells in each group (scale bar = 25 μm); (F) Colony formation assay detecting the colony formation ability of SW480 cells in each group; (G) Flow cytometry detecting cell cycle changes in SW480 cells in each group; (H) Western blot detecting the expression changes of cell cycle-related proteins in SW480 cells in each group; (I) Glucose uptake in SW480 cells in each group; (J) Lactate production in SW480 cells in each group; (K) Western blot detecting the expression of glycolytic rate-limiting enzymes in SW480 cells in each group. * represents a difference compared to the oe-NC or PBS group (P < 0.05), # represents a difference compared to the LEVs+oe-NC group (P < 0.05), experiments repeated 3 times. (JPG 1779 KB) [file 10565_2024_9897_MOESM2_ESM.jpg]

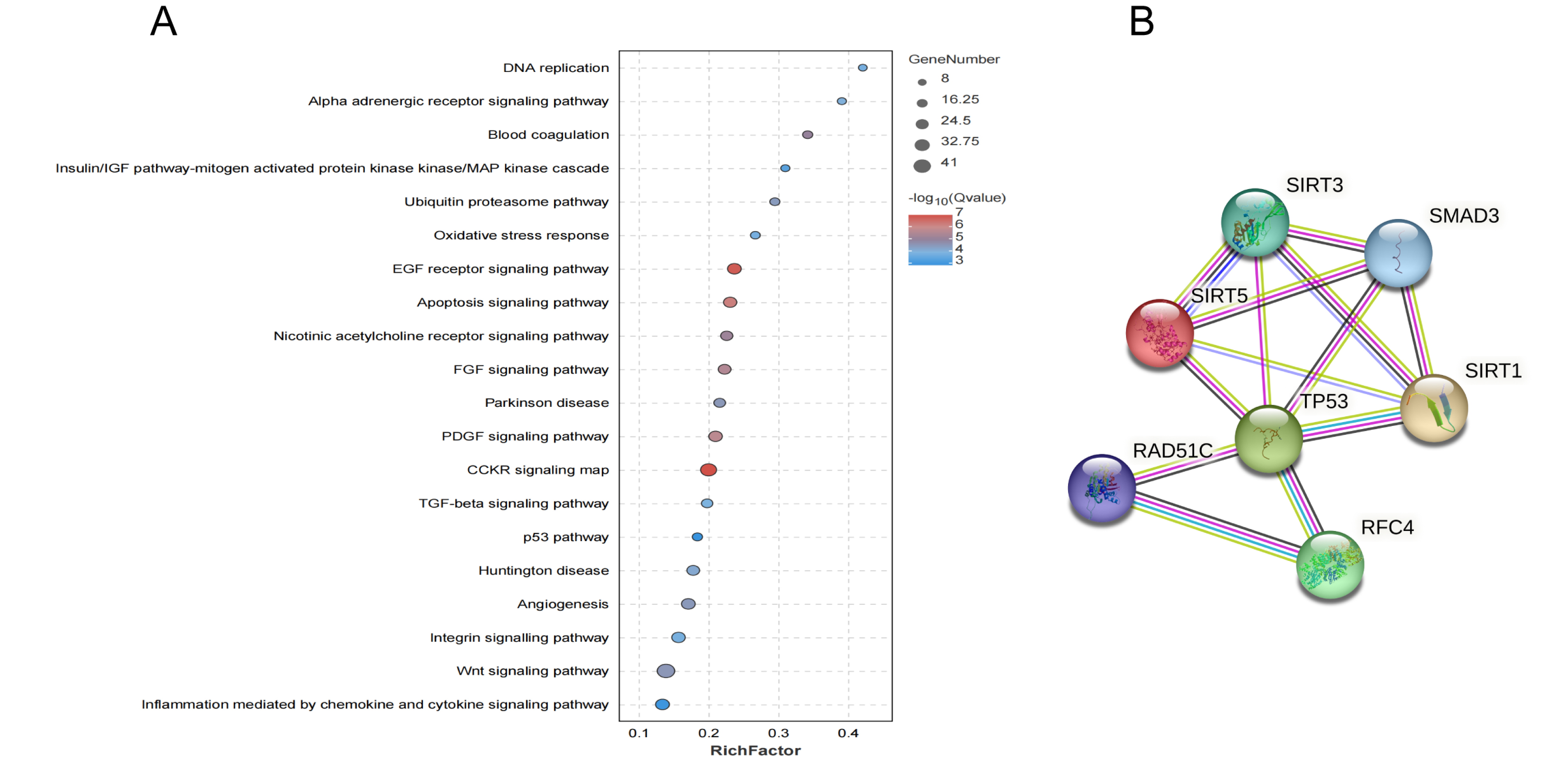

Supplement: Supplementary file 3 — Supplementary file3 Figure S3. Interactions between SIRT5 and p53. Note: (A) KEGG pathway enrichment analysis of differentially expressed genes obtained from Caco-2 cells treated with LEVs; (B) Protein interaction network of candidate target genes encoded by p53 (TP53) and their encoded proteins. (JPG 1588 KB) [file 10565_2024_9897_MOESM3_ESM.jpg]

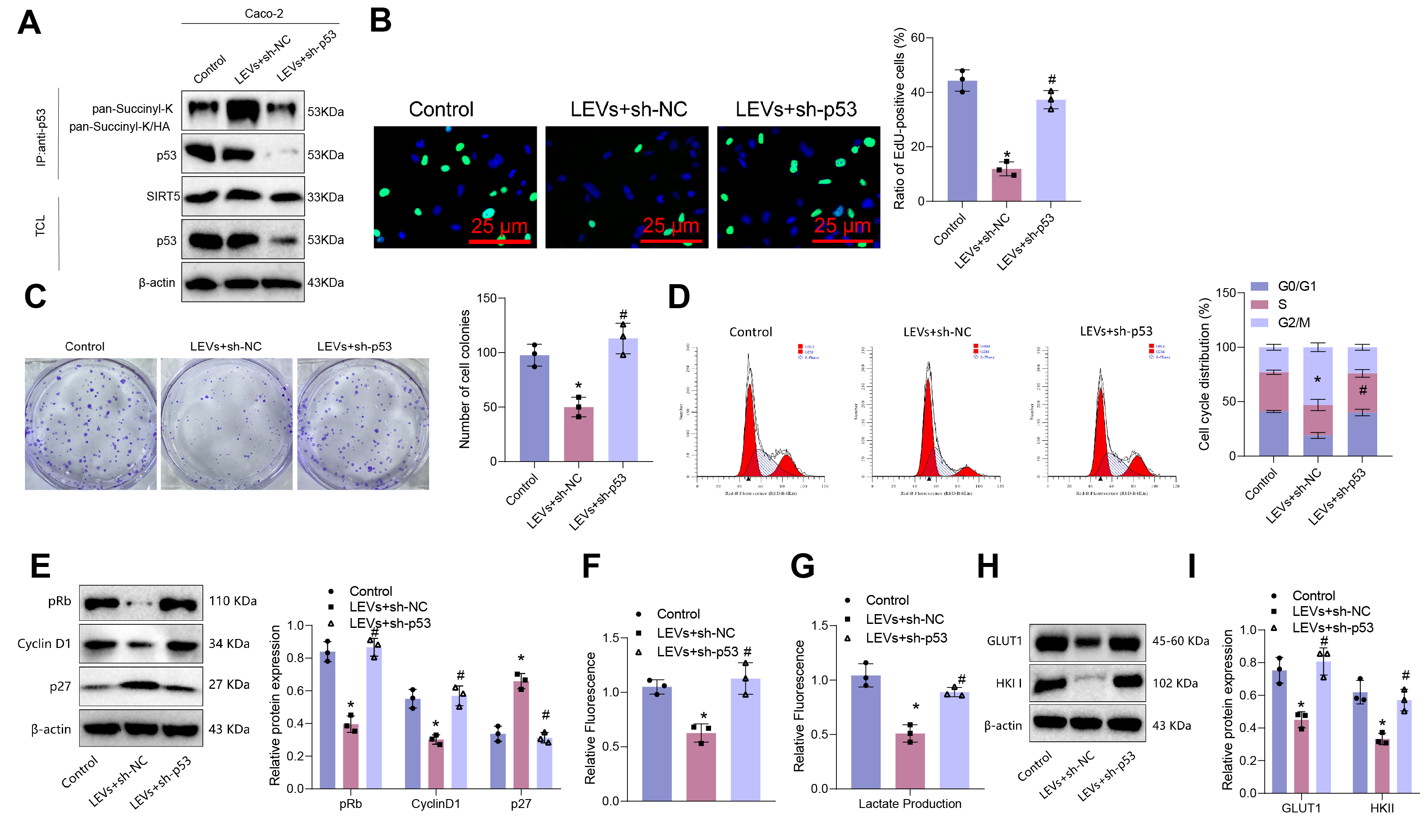

Supplement: Supplementary file 4 — Supplementary file4 Figure S4. LEVs regulate the SIRT5/p53 axis, affecting the proliferation and glycolysis metabolism of colorectal cancer cells SW480. Note: (A) co-IP experiment detecting the level of acetylated p53 protein in different groups of SW480 cells; (B) EdU staining detecting the proliferation of SW480 cells in each group (scale bar = 25 μm); (C) Colony formation assay detecting the colony formation ability of SW480 cells in each group; (D) Flow cytometry detecting cell cycle changes in SW480 cells in each group; (E) Western blot detecting the expression changes of cell cycle-related proteins in SW480 cells in each group; (F) Glucose uptake in SW480 cells in each group; (G) Lactate production in SW480 cells in each group; (H-I) Western blot detecting the expression of glycolytic rate-limiting enzymes in SW480 cells in each group. * represents a difference compared to the Control group (P < 0.05), # represents a difference compared to the LEVs+sh-NC group (P < 0.05), experiments repeated 3 times. (JPG 1177 KB) [file 10565_2024_9897_MOESM4_ESM.jpg]

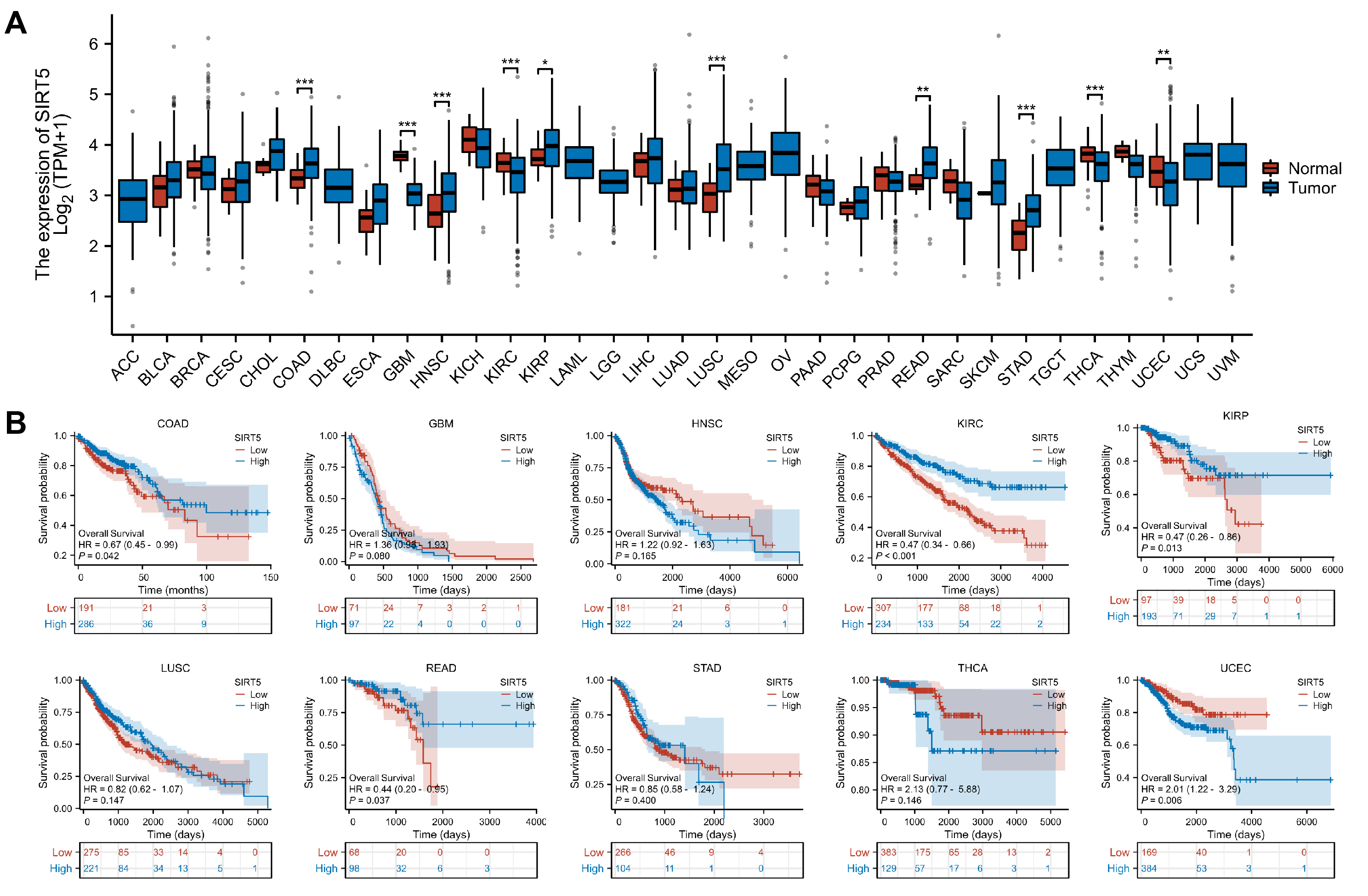

Supplement: Supplementary file 5 — Supplementary file5 Figure S5. Expression Levels of SIRT5 in Other Cancers and Its Correlation with Prognosis. Note: (A) SIRT5 expression levels in 33 cancer tissues and normal tissues; (B) Protein expression of SIRT5 and survival curve analysis of patients with COAD, GBM, HNSC, KIRC, KIRP, LUSC, READ, STAD, THCA, and UCEC; * indicates P < 0.05 compared to the Normal group; ** indicates P < 0.01 compared to the Normal group; *** indicates P < 0.001 compared to the Normal group. (JPG 1330 KB) [file 10565_2024_9897_MOESM5_ESM.jpg]

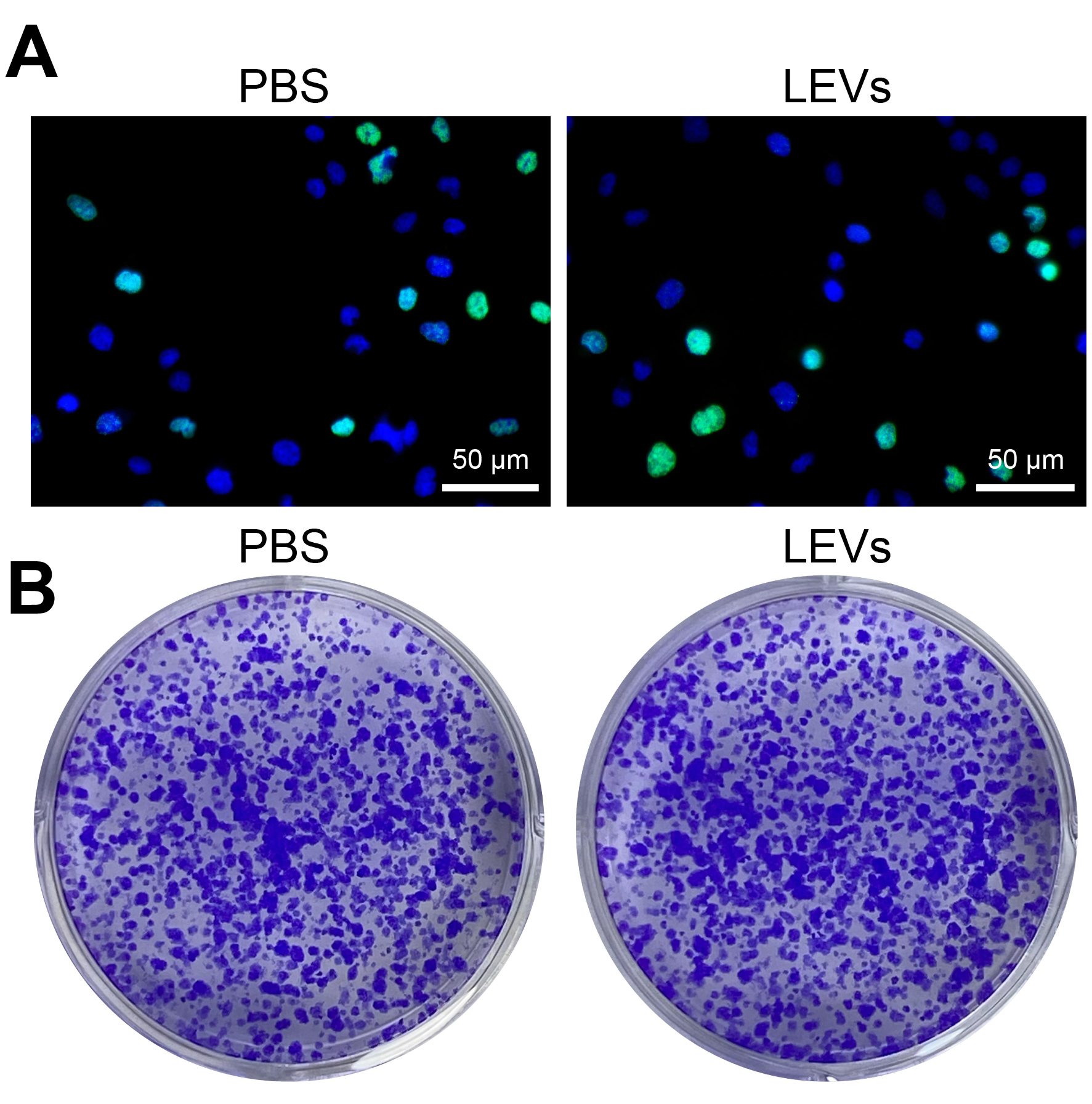

Supplement: Supplementary file 6 — Supplementary file6 Figure S6 LEVs inhibit NCM460 cell growth. NCM460 cells were treated with isolated LEVs, followed by (A) EdU staining to assess proliferation of various groups of Caco-2 cells (scale bar=25 μm); (B) colony formation assay to evaluate the clonogenic ability of different groups of Caco-2 cells, with the cell experiment repeated three times. (JPG 1145 KB) [file 10565_2024_9897_MOESM6_ESM.jpg]
