# Supplementary material for: Antitumorigenic potential of Lactobacillus-derived extracellular vesicles: p53 succinylation and glycolytic reprogramming in intestinal epithelial cells via SIRT5 modulation
Source: Cell Biol Toxicol. 2024 Aug 7;40(1):66. doi: 10.1007/s10565-024-09897-y (PMC11306434; doi:10.1007/s10565-024-09897-y)
Supplement: Supplementary file 7 — Supplementary file7 (DOCX 12 KB) [file 10565_2024_9897_MOESM7_ESM.docx]

**Table S1. qRT-PCR primer sequences**

| Gene (human) | primer sequences |
| --- | --- |
| SIRT5 | F 5' -CGTGGTCATCACCCAGAACA-3' |
|  | R 5' -AGCCACAACTCCACAAGAGG-3' |
| RAD51C | F 5' -GCATTAGGGGAAAGTTGGGGA-3' |
|  | R 5' -TGCCAACCTTTGCTTTCGG-3' |
| RFC4 | F 5' -TTGGGCCTGAACTTTTCCGA-3' |
|  | R 5' -GCTTCCCATCTGAGCGACTT-3' |
| SIRT1 | F 5' -TTGGGTACCGAGATAACCTTCTG-3' |
|  | R 5' -TGTTCGAGGATCTGTGCCAATCA-3' |
| SMAD3 | F 5' -TCGTCCATCCTGCCTTTCAC-3' |
|  | R 5' -CTGCCCCGTCTTCTTGAGTT-3' |
| TP53 (p53) | F 5' -GCGACCTATGGAAACTACTTCCTG-3' |
|  | R 5' -CTGGCATTCTGGGAGCTTCA-3' |
| β-actin | F 5′-AATTTGCGTGTGGCTCCCGAGG-3′ |
|  | R 5′-GGATAGCACAGCCTGGATAGCA-3′ |
